# MicroRNA-877-5p Inhibits Cell Progression by Targeting FOXM1 in Lung Cancer

**DOI:** 10.1155/2022/4256172

**Published:** 2022-06-15

**Authors:** Zhiguang Liu, Xinlian Wang, Liqiang Cao, Xiaowei Yin, Qian Zhang, Lan Wang

**Affiliations:** ^1^Department of Respiratory and Critical Care Medicine, Changzhou Second People's Hospital Affiliated to Nanjing Medical University, Changzhou 213164, Jiangsu, China; ^2^Department of Thoracic Surgery, Wuxi Branch of Zhongda Hospital Affiliated to Southeast University, Wuxi 214105, Jiangsu, China; ^3^Department of Respiratory and Critical Care Medicine, The Jiangyin Clinical College of Xuzhou Medical University, Jiangyin 214400, Jiangsu, China

## Abstract

**Background:**

Many researches revealed that microRNAs (miRNAs) function as potential oncogene or tumor suppressor gene. As an antioncogene, miR-877-5p was reduced in many tumors.

**Objective:**

This research aimed to explore the biological role and mechanisms of miR-877-5p, which may help patients with non-small-cell lung cancer (NSCLC) find effective therapeutic targets.

**Methods:**

The downstream targets of miR-877-5p were predicted by Bioinformatics software. RT-qPCR and western blot were employed to analyze the gene levels. The impacts of miR-877-5p and FOXM1 were assessed by cell function experiments.

**Results:**

The miR-877-5p was reduced in NSCLC. In addition to this, it also inhibited cell progression of NSCLC cells *in vitro*. Moreover, the upregulation of FOXM1 expression restored the inhibitory effect of enhancement of miR-877-5p.

**Conclusions:**

Taken together, miR-877-5p inhibited cell progression by directly targeting FOXM1, which may provide potential biomarkers for targeted therapy of NSCLC.

## 1. Introduction

Recently, cases rapidly increase in cancer incidence and mortality worldwide. In both sexes, lung cancer is a prevalent cancer (approximately 11.6%) and the main reason of cancer deaths (approximately18.4%) [1]. Non-small-cell lung cancer (NSCLC), as a common type, has a five-year survival rate of only approximately 15%. Since the majority of patients are in advanced or metastatic stage, the prognosis is very poor [2]. In addition, the specificity of chemotherapy is weak, so the adverse reactions associated with the treatment are dramatically strong [3]. Compared with traditional chemotherapy, precision therapy effectively improves the treatment outcomes [4]. However, targeted therapy still inevitably produces drug resistance and has certain limitations. Hence, it is necessary to investigate the potential mechanisms to identify new underlying biomarkers for NSCLC.

MicroRNA (miRNA) is approximately 22 nucleotides in length, with the function of regulating posttranscriptional genes in cells [5]. Specifically, this function works by binding to the 3′-UTR of the target messenger RNA (mRNA) [6]. According to reports, miRNAs are related to the development of a variety of diseases, and they may be used as diagnostic biomarkers [7]. Among them, cancer has been a major focus of miRNA research [8]. Many miRNAs are also participated in the regulation of genes in cancer, showing the activity of inhibiting or promoting tumor activity [9]. MicroRNA expression profiles show that the dysregulation of miRNA molecules or miRNA clusters have major impacts on the progression of cancer, containing NSCLC [10, 11].

miR-27a regulated the Wnt/*β*-catenin axis by targeting SFRP1 to promote the development of cells in colon cancer [12]. miR-22 targets NLRP3 and inhibits cell progression in colorectal cancer [13]. miR-143 overexpression suppressed cell proliferation in CAMA-1 cells [14]. miR-16-5p inhibited cell processes via regulating AKT3 in prostate cancer [15]. miR-134 and miR-218-5p overexpression can inhibit NSCLC progression via targeting EGFR [16, 17]. Consequently, miRNAs associated with NSCLC still need further research, which may provide patients with new promising therapeutic targets.

It was found that miR-877-5p acts as a tumor inhibitor in the regulation of a variety of cancers, for example, liver cancer [18], cervical cancer [19], laryngeal squamous cell carcinoma [20], and gastric cancer [21]. However, there are few reports about miR-877-5p in NSCLC. The important mechanisms in the tumorigenesis and progression of NSCLC need to be further explored. Therefore, this study intended to study the effects and potential mechanism of miR-877-5p in NSCLC.

This study demonstrated that miR-877-5p was obviously reduced in NSCLC. According to cell function experiments, we found that miR-877-5p inhibited the progression of NSCLC cell lines. Apart from that, FOXM1 was predicted by bioinformatics software as a downstream target of miR-877-5p. The data revealed that miR-877-5p suppressed the tumorigenesis and development of NSCLC by targeting FOXM1.

## 2. Materials and Methods

### 2.1. Clinical Samples

The experimental protocols were approved by the Ethics Committee of the Changzhou Second Affiliated People's Hospital of Nanjing Medical (Jiangsu, China). Patients were required to sign written informed consent before participating in the research. 37 pairs of tumor and nontumor tissues were collected from NSCLC patients.

### 2.2. Cell Culture

BEAS-2B, NCI-H661, NCI-H460, A549, and NCI-H1299 were obtained from the Shanghai Institute of Biochemistry and Cell Biology. RPMI-1640 containing 10% FBS, 1% penicillin G, and streptomycin (all from Gibco) was employed to culture cells at 37°C with 5% CO_2_.

### 2.3. Transient Transfection

The miR-877-5p mimics and miR-NC mimics were obtained from GenePharma (Shanghai, China). The siRNA targeting FOXM1 (si-FOXM1), si-NC, FOXM1 overexpression plasmid (pcDNA-FOXM1), and empty vector pcDNA were synthesized by RiboBio (Guangzhou, China). In short, cells (5 × 10^5^/well) were planted into 6-well plates. Cells were transfected with 100 pmol oligonucleotides or 4 *µ*g plasmid by Lipofectamine®2000 (Invitrogen). Then the cells with 48 h transfection were collected. RT-qPCR was conducted to assay transfected efficacy.

### 2.4. RT-qPCR

Total RNA was extracted from NSCLC tissue or cells using the TRIzol® reagent (Invitrogen). TaqMan® MicroRNA Reverse Transcription kits (Applied Biosystems) were employed to generate cDNA. BeyoFast™ SYBR Green qPCR Mix (Beyotime) was performed to carry out RT-qPCR in an ABI Prism 7500 Sequence Detection System. U6 and GAPDH were employed to normalize the gene levels. The 2^−ΔΔCt^ method was performed to calculate gene levels. The primer sequence was shown as follows (5ʹ-3ʹ): miR-877-5p F: TAGAGGAGATGGCGCAG; R: GAACATGTCTGCGTATCTC; FOXM1 F: AGCAGTCTCTTACCTTCC; R: CTGGCAGTCTCTGGATAA; GAPDH F: GCAACTAGGATGGTGTGGCT; R: TCCCATTCCCCAGCTCTCATA; U6 F: AAAGCAAATCATCGGACGACC; R: GTACAACCATTGTTTCCTCGGA.

### 2.5. CCK-8 Assay

CCK-8 kit (Dojindo Molecular Technologies) was carried out to assay proliferation. Briefly, cells (3 × 10^4^) were seeded in 96-well plates. Then, CCK-8 regent (10 *μ*L) was supplemented after 0, 24, 48, and 72 hours of incubation. Next, the cells were continued incubating for 2 h. Finally, OD450 was assayed to evaluate the ability of proliferation.

### 2.6. Transwell Assay

The transwell chamber (8 *µ*m, BD Biosciences) without or with Matrigel (Sigma-Aldrich) was employed to assay the ability of cell migration or invasion, respectively. Then 5 × 10^4^ cells in 200 *µ*l RPMI without FBS were supplemented to the upside compartment, and a total of 560 *µ*l RPMI with 15% FBS was supplemented to the lower compartments. After incubation for 24 h, the upper side cells were discarded. The cells in the bottom chamber were fixed and stained with 4% paraformaldehyde and 0.1% crystal violet, respectively. The number of migratory or invasive cells was counted and photographed by selecting five random fields.

### 2.7. Dual-Luciferase Reporter Assay

A wild-type (WT) and mutant-type (MUT) FOXM1 sequence fragments were cloned into the pmirGLO plasmid (Promega). In short, cells (1 × 10^5^/well) were seeded into 24-well plates for 24 hours. Then, miR-877-5p or miR-NC and pmirGLO-FOXM1-WT or pmirGLO-FOXM1-MUT were cotransfected into cells using Lipofectamine®2000. After 48 h cotransfection, cells were collected and a dual-luciferase reporter assay system (Promega) was adopted to evaluate luciferase activity. The Ranilla luciferase activity was employed as an internal control.

### 2.8. Western Blotting

RIPA lysis buffer was employed to extract total proteins of cells (Beyotime). A BCA Protein Quantification kit was devoted to evaluate the protein concentration (Beyotime). Then, the protein was separated using 10% SDS-PAGE. Next, separated proteins were transferred onto PVDF membranes (Millipore). Next, the membranes were blocked for 2 h in TBST containing 5% skimmed milk. Then the membranes were incubated with primary antibody (anti-FOXM1: ab207298 or anti-GAPDH: ab9485, 1 : 1000, Abcam) at 4°C overnight. After washing three times with TBST, the membranes were further incubated with HRP-conjugated secondary antibody (1 : 10,000; ab205718; Abcam) for 2 h. GAPDH was employed as the internal control. The protein bands were visualized by an enhanced chemiluminescence (ECL) system (EMD Millipore). ImageJ was used to analyze the protein signals.

### 2.9. In Vivo Tumorigenesis

BALB/c nude mice (12 females; 4-5 weeks; 15–20 g; *n* = 6) were purchased from the Vital River company (Beijing, China). The mice were divided into two groups at random, and A549 cells (2 × 10^7^) transfected with miR-877-5p mimics or miR-NC mimics were inoculated subcutaneously on the left side, and a vernier caliper was devoted to measure the length (L) and width (W) every 3 d. The formula L × W^2^ × 0.5 mm^3^ was conducted to evaluate the volume of tumors. The mice were euthanized after 18 d of A549 cells injection. Tumor tissues were then excised, weighed, and collected to further analyze the gene expression. These *in vivo* experiments were approved by the Animal Ethics Committee of Changzhou Second Affiliated People's Hospital of Nanjing Medical.

### 2.10. Statistical Analysis

All experiments data in this study were presented as means ± standard deviation (SD) with at least thrice independent experiments. All statistical analyses were conducted through GraphPad Prism 8.0. Student's *t*-tests were performed for comparisons between two samples. The correlations between miR-877-5p and FOXM1 mRNA levels were evaluated by Spearman's correlation analysis. *P* < 0.05 was regarded as a significant difference.

## 3. Results

### 3.1. miR-877-5p Is Suppressed in NSCLC

**Figure 1:**
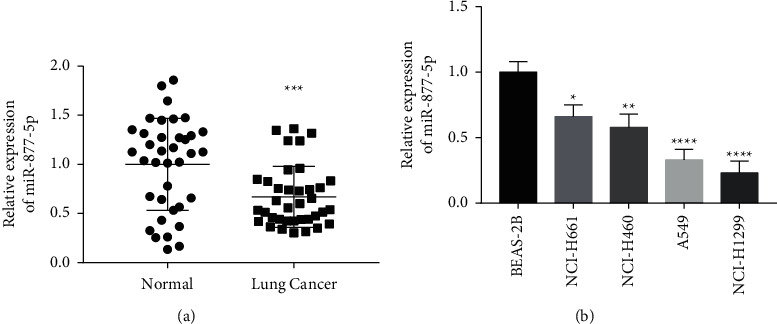
The expression of miR-877-5p in NSCLC tissues and cell lines. (a) RT-qPCR analyses of the expression levels of miR-877-5p in 37 pairs of NSCLC tissues and adjacent normal tissues. (b) Expression levels of miR-877-5p were detected by RT-qPCR in four NSCLC cell lines (NCI-H661, NCI-H460, A549, and NCI-H1299) and a nontumorigenic bronchial epithelium cell line (BEAS-2B). ^*∗*^*P* < 0.05, ^*∗∗*^*P* < 0.01, ^*∗∗∗*^*P* < 0.001 and ^*∗∗∗∗*^*P* < 0.0001 vs. normal tissues/BEAS-2B.

Firstly, 37 pairs of tumor and nontumor tissues were evaluated to identify the endogenous miR-877-5p expression in NSCLC. The data showed that miR-877-5p was obviously suppressed in NSCLC (Figure 1(a)). Then, The Cancer Genome Atlas (TCGA) was employed to analyze the expression of miR-877-5p in lung cancer. The results confirmed that miR-877-5p was obviously reduced in lung cancer (Figure S1 and Excel S1). Additionally, we also confirmed miR-877-5p expression in four NSCLC cell lines, including NCI-H661, NCI-H460, A549, and NCI-H1299. As expected, miR-877-5p was noticeably reduced in NSCLC cells (Figure 1(b)). Among these, we chose A549 and NCI-H1299, which have the lowest expression level of miR-877-5p, for follow-up experiments. These data suggested that miR-877-5p may be involved in the carcinogenesis and progression of NSCLC.

### 3.2. Enhancement of miR-877-5p Inhibits the NSCLC Cell Process

**Figure 2:**
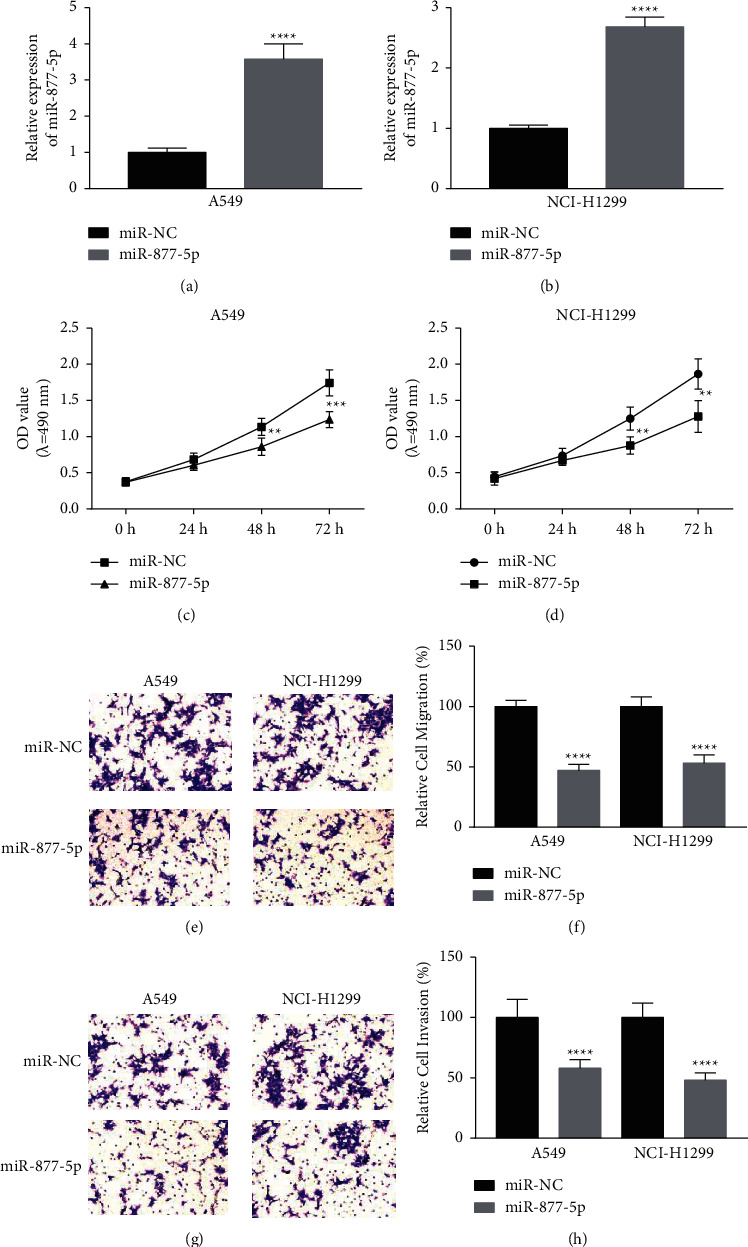
Overexpression of miR-877-5p regulates the proliferation, migration, and invasion of A549 and NCI-H1299 cells. miR-877-5p mimics or miR-NC were transfected into A549 (a) and NCI-H1299 (b) cells. The proliferative ability of A549 (c) and NCI-H1299 (d) cells was assayed by CCK-8. Transwell was performed to measure the migration of A549 (e) and NCI-H1299 (f) cells (magnification, x200). Transwell invasion assays of A549 (g) and NCI-H1299 (h) cells (magnification, x200). ^*∗*^*P* < 0.05, ^*∗∗*^*P* < 0.01, ^*∗∗∗*^*P* < 0.001 and ^*∗∗∗∗*^*P* < 0.0001 vs. miR-NC.

Next, A549 and NCI-H1299 cells were transfected with miR-877-5p mimics to explore the potential functions of miR-877-5p in NSCLC. RT-qPCR results confirmed that miR-877-5p was notably induced in A549 and NCI-H1299 cells transfected with miR-877-5p mimics (Figure 2(a) and 2(b)). CCK-8 assay results revealed that the introduction of miR-877-5p obviously attenuated cell proliferation in NSCLC cells (Figures 2(c) and 2(d)). Afterwards, transwell data demonstrated that enhancement of miR-877-5p significantly reduced the migration (Figures 2(e) and 2(f)) and invasion (Figures 2(g) and 2(h)) in A549 and NCI-H1299. The data confirmed that miR-877-5p inhibited cell progression by acting as an inhibitor gene in NSCLC.

### 3.3. miR-877-5p Directly Targets FOXM1 in NSCLC Cells

**Figure 3:**
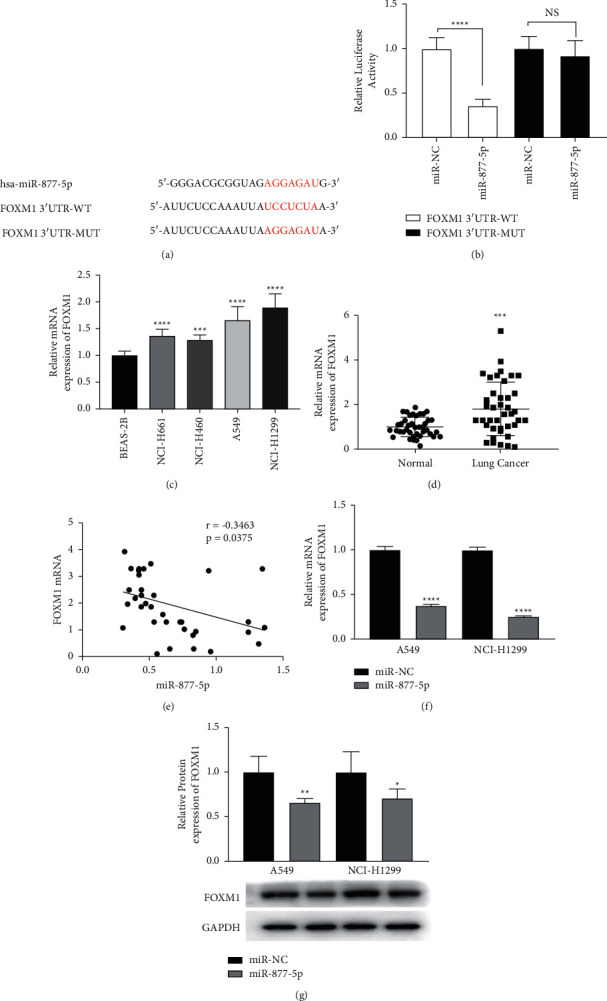
FOXM1 is a direct downstream gene of miR-877-5p. (a) The predicted binding sequence of miR-877-5p and FOXM1. (b) Luciferase reporter assay was conducted to verify the relationship between miR-877-5p and FOXM1. (c) Expression levels of FOXM1 were detected in cells. (d) The mRNA levels of FOXM1 in tissues. (e) Spearman's correlation scatter plot. *r* = −0.3463, *P*=0.0375. The mRNA (f) and protein (g) expression of FOXM1 in A549 and NCI-H1299 cells. ^*∗*^*P* < 0.05, ^*∗∗*^*P* < 0.01, ^*∗∗∗*^*P* < 0.001, and ^*∗∗∗∗*^*P* < 0.0001.

In order to explore the underlying mechanisms of miR-877-5p in the proliferation and metastasis of NSCLC, we next tried to determine the potential molecular targets of miR-877-5p by bioinformatics. By cross-analysing predicted targets from five databases (miRDIP, TargetScan, Starbase, miRDB, and miRTarBase), we identified a total of 5 common targets (FOXM1, SORBS3, CDKN1B, KRAS, and ZNF174) and mapped Venn diagrams (Figure S2 and Excel S2). We selected FOXM1 for subsequent analysis and predicted its binding site to miR-877-5p (Figure 3(a)). T (Figure 3(a)). Subsequently, the relationship between FOXM1 and miR-877-5p was verified by a dual-luciferase reporter assay. We found that miR-877-5p mimics remarkably downregulated the luciferase activity in WT-FOXM1 3′-UTR group (Figure 3(b)), indicating that FOXM1 and miR-877-5p have a target-binding relationship. Next, the FOXM1 mRNA expression in NSCLC were elucidated. The investigation of the expression of FOXM1 in NSCLC cells showed great upregulation of FOXM1 in all the NSCLC cells (Figure 3(c)). The expression of FOXM1 mRNA was also markedly up-regulated in NSCLC tissues (Figure 3(d)). Finally, we assessed whether miR-877-5p regulated the expression of FOXM1. We observed that miR-877-5p was negatively correlated with FOXM1 expression (*p*=0.0375; *r* = −0.3463; Figure 3(e)). Furthermore, the data revealed that FOXM1 expression was markedly downregulated in the A549 and NCI-H1299 cells transfected with miR-877-5p mimics (Figure 3(f)). Western blot showed a similar result (Figure 3(g)). These results indicated that miR-877-5p directly targeted and negatively regulated the expression of FOXM1 in NSCLC.

### 3.4. Reduction of FOXM1 Attenuates the NSCLC Cell Process

**Figure 4:**
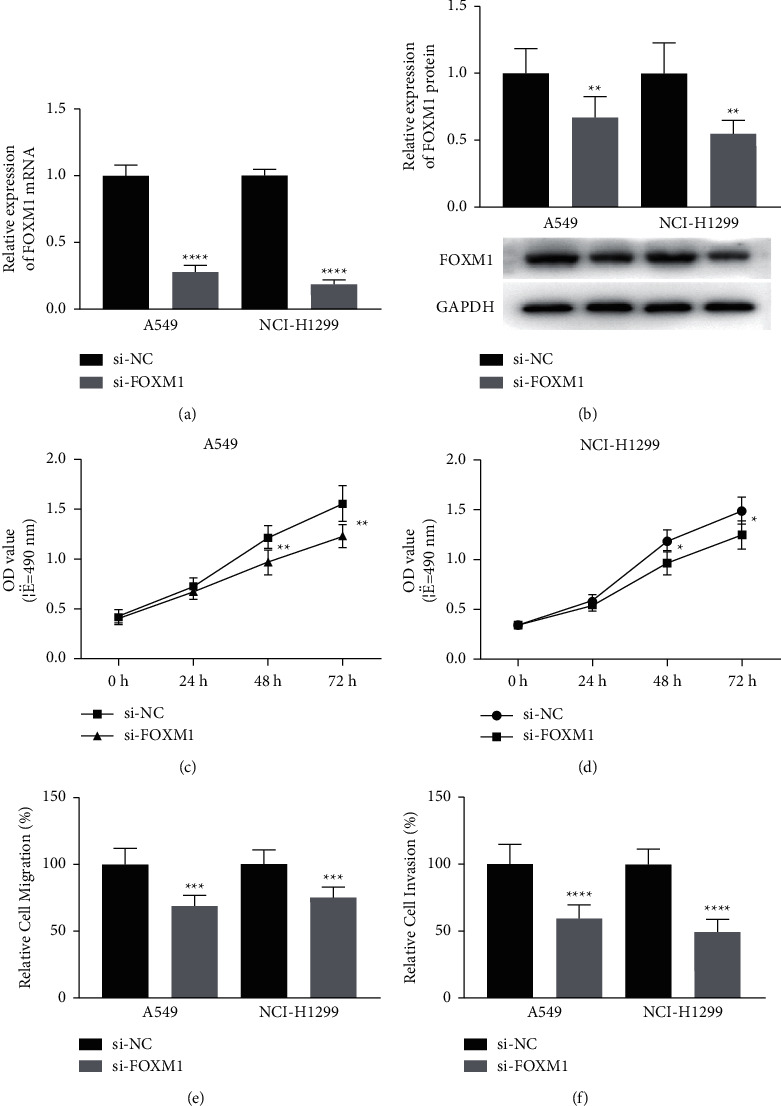
Knockdown of FOXM1 inhibits the progression of NSCLC cells. A549 and NCI-H1299 cells transfected with si-NC or si-FOXM1, respectively. (a) FOXM1 mRNA levels were assayed in transfected A549 and NCI-H1299 cells. (b) FOXM1 protein levels were evaluated in transfected A549 and NCI-H1299 cells. The proliferation abilities of transfected A549 (c) and NCI-H1299 cells (d). (e) Migration assays of transfected A549 and NCI-H1299 cells. (f) Invasion assays of transfected A549 and NCI-H1299 cells. ^*∗*^*P* < 0.05, ^*∗∗*^*P* < 0.01, ^*∗∗∗*^*P* < 0.001, and ^*∗∗∗∗*^*P* < 0.0001.

To further explore the functions of FOXM1 in NSCLC, cells were transfected with si-FOXM1. We observed that mRNA (Figure 4(a)) and protein (Figure 4(b)) expression of FOXM1 were notably decreased after FOXM1 si-RNA transfection. Furthermore, CCK-8 and transwell results indicated that knockdown of FOXM1 alleviated cell proliferation (Figure 4(c) and 4(d)), migration (Figure 4(e)), and invasion (Figure 4(f)). Therefore, the data further indicate that reduction of FOXM1 inhibited cell progression in NSCLC.

### 3.5. FOXM1 Antagonizes the Inhibitory Effect of miR-877-5p

**Figure 5:**
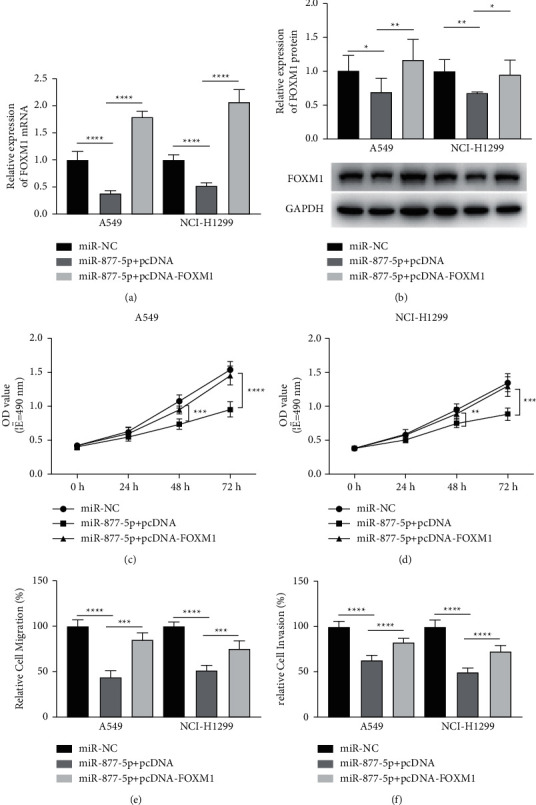
Restoration of FOXM1 expression reverses the antitumor effect of miR-877-5p in NSCLC cells. A549 and NCI-H1299 cells transfected with miR-NC, miR-877-5p + pcDNA, or miR-877-5p + pcDNA-FOXM1, respectively. (a) The expression of FOXM1 mRNA in transfected A549 and NCI-H1299 cells. (b) Protein levels of FOXM1 in transfected A549 and NCI-H1299 cells. The proliferation of transfected A549 (c) and NCI-H1299 (d) cells. (e) Migration assays of transfected A549 and NCI-H1299 cells. (f) Invasion assays of transfected A549 and NCI-H1299 cells. ^*∗*^*P* < 0.05, ^*∗∗*^*P* < 0.01, ^*∗∗∗*^*P* < 0.001, and ^*∗∗∗∗*^*P* < 0.0001.

Rescue experiments were employed in order to further confirm the mechanism of miR-877-5p axis. Cells were cotransfected with FOXM1 in combination with or without miR-877-5p. Following transfection, the expression of FOXM1 were obviously reduced in miR-877-5p overexpression cells; however, the decreased FOXM1 expression was restored following pcDNA- FOXM1 cotransfection in A549 and NCI-H1299 cells (Figures 5(a) and 5(b)). We further observed that restoration of FOXM1 partially rescued the growth of A549 (Figure 5(c)) and NCI-H1299 (Figure 5(d)) which were reduced via miR-877-5p. Furthermore, transwell cell migration (Figure 5(e)) and invasion (Figure 5(f)) assays demonstrated that overexpression of FOXM1 eliminated the inhibitory impacts by miR-877-5p overexpression. Collectively, rescue experiments data revealed that miR-877-5p inhibited the progression of NSCLC by targeting FOXM1.

### 3.6. miR-877-5p Overexpression Attenuates Tumor Growth In Vivo

**Figure 6:**
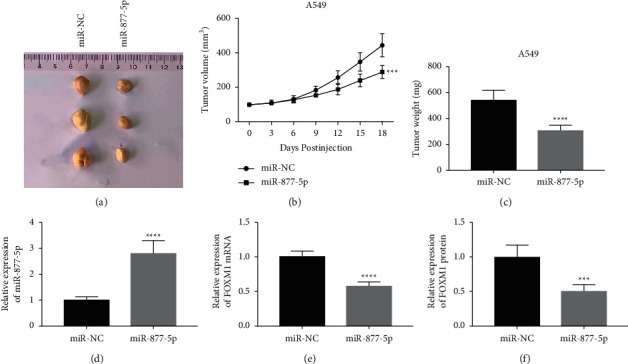
miR-877-5p overexpression' suppresses tumorigenesis of NSCLC *in vivo*. (a) Representative images of tumor tissues. (b) The tumor growth curve. (c) The mice were killed and tumor tissue was weighed on day 18. (d) miR-877-5p expression in the tumors. The mRNA (e) and protein (f) expression of FOXM1 in the tumors. ^*∗*^*P* < 0.05, ^*∗∗*^*P* < 0.01, ^*∗∗∗*^*P* < 0.001, and ^*∗∗∗∗*^*P* < 0.0001.

Finally, we further investigated whether miR-877-5p could inhibits the tumor growth of NSCLC in the transplanted tumor model. The integrated tumor growth curve revealed that the growth of tumors treated with the miR-877-5p was obviously inhibited (Figure 6(a)). Consistently, miR-877-5p overexpression was obviously decreased the weight of tumor (Figure 6(b)). RT-qPCR detection showed that compared with the miR-NC group, miR-877-5p was remarkably increased after miR-877-5p introduction (Figure 6(c)). Furthermore, the data demonstrated that FOXM1 mRNA and protein expressions were also markedly decreased in the miR-877-5p group (Figures 6(d) and 6(e)). In summary, our data manifested that miR-877-5p attenuated the tumorigenicity of NSCLC cells *in vivo*.

## 4. Discussion

MicroRNAs are involved in the regulation of various cellular processes, which indicates that they may be a class of promising biomarkers for treatment and prognosis [22]. In recent years, the roles of miRNAs in NSCLC have been emphasized [23]. miR-130b overexpression promotes the lung cancer cell progression by PPAR*γ*/VEGF-A/BCL-2 [24]. Overexpressed miR-33b inhibits glucose metabolism by acting as an anti-NSCLC molecule in NSCLC cells [25]. MicroRNA-877 inhibits the development of NSCLC by regulating IGF-1R [26]. miR-641 overexpression attenuated the cell proliferation by regulating MDM2 and p53 to induce apoptosis in A549 cells [27]. Moreover, many studies evidenced that miR-877 family members are reduced in many human malignant tumors. miR-877 was reduced and alleviated cell proliferation by MACC1 in cervical cancer [28]. miR-877 inhibited the ability of proliferation by blocking G1/S phase in liver cancer [29]. miR-877-3p overexpression alleviates cell proliferation by blocking the G1 phase in bladder cancer cells [30]. miR-877 inhibits cell progression by downregulating AQP3 in gastric cancer [31]. All of the above cleared that miR-877 family are important indicators and promising therapeutic targets in human malignant tumors.

This study detected miR-877-5p levels in NSCLC and explored the effects and mechanism of miR-877-5p in NSCLC. According to the analysis of RT-qPCR, we observed that miR-877-5p was obviously reduced in NSCLC. It means that miR-877-5p may involve in cell progression of NSCLC. Subsequently, cell function experiments revealed that miR-877-5p overexpression blocked cell proliferation, migration, and invasion in NSCLC. This suggested that miR-877-5p played an inhibitor role in NSCLC. The mechanism of miR-877-5p remains unclear yet. It is well known that miRNAs work by directly regulating their targeted mRNA [32]. Herein, bioinformatics software was employed to predict the directly binding genes of miR-877-5p and finally proved that FOXM1 was a directly target gene through dual luciferase reporter gene analysis.

The forkhead box protein M1 (FOXM1), as a member of the forkhead transcription factor family, plays an important role in many kinds of cell processes [33]. FOXM1 also may act as both direct and indirect targets for tumor therapeutic intervention [34]. Abnormal upregulation of FOXM1 is related to the development of the majority of human cancers, such as bladder cancer [35], NSCLC [36], colorectal cancer [37], cervical cancer [38], and hepatocellular carcinoma [39]. It has been reported that miR-216b inhibited cell proliferation by FOXM1 in cervical cancer [40]. miR-320 inhibited the progression of HCT-116 cells and enhanced the sensitivity of cells to chemotherapy by targeting FOXM1 [41]. miR-134 inhibited EMT by regulating FOXM1 in NSCLC cells [42]. lncRNA MFI2-AS1 promotes hepatocellular carcinoma progression by the miR-134/FOXM1 axis [43]. FOXM1 also promoted progression of gastric cancer by synergistically with PLAU [44]. These studies suggested that the abnormal expression of FOXM1 may be a common feature of many cancers, and targeting FOXM1 may provide a new indicator of treatment strategies and cancer prognosis.

In this paper, we found that FOXM1 was increased in NSCLC. Enhancement of miR-877-5p significantly inhibited FOXM1 expression levels. In addition, miR-877-5p was negatively correlated with FOXM1 expression. In addition, FOXM1 knockdown significantly inhibited cell progression. Subsequently, we conducted rescue experiments to verify the anticancer effect of miR-877-5p/FOXM1 in NSCLC. As expected, our data evidenced that reintroduction of FOXM1 significantly reversed the suppressive impacts of miR-877-5p on the cell aggressiveness. These data indicated that miR-877-5p exerted its inhibitory effects of cancer by negatively regulating FOXM1. Most importantly, miR-877-5p overexpression limited tumor growth *in vivo*.

Overall, our research cleared that miR-877-5p was dysregulated and acted as a tumor suppressor in NSCLC. miR-877-5p overexpression alleviated NSCLC cell progression *in vitro* and restricted the growth of xenogeneic tumors *in vivo*. Moreover, FOXM1 was upregulated and proved to be a downstream mRNA of miR-877-5p. The miR-877-5p/FOXM1 represented a new pathway to regulate the development of NSCLC, which may provide a potential target for the treatment of NSCLC.

## Data Availability

The data used to support the findings of this study are available from the corresponding author upon request.

## Supplementary Materials

Supplementary Materials This section provides additional information about the expression of miR-877-5p in the TCGA publicly available lung cancer dataset (Supplementary Figure S1 and Supplementary Excel S1), targets, and Venn diagrams of miR-877-5p predicted by five databases cross-analysing (Supplementary Figure S2 and Supplementary Excel S2).
