# Supplementary material for: MicroRNA-877-5p Inhibits Cell Progression by Targeting FOXM1 in Lung Cancer
Source: Can Respir J. 2022 Jun 15;2022:4256172. doi: 10.1155/2022/4256172 (PMC9217556; doi:10.1155/2022/4256172)
Supplement: Supplementary Materials — This section provides additional information about the expression of miR-877-5p in the TCGA publicly available lung cancer dataset (Supplementary Figure S1 and Supplementary Excel S1), targets, and Venn diagrams of miR-877-5p predicted by five databases cross-analysing (Supplementary Figure S2 and Supplementary Excel S2). [file 4256172.f1.zip › 4256172.f1/Supplement Excle S1.pdf]

<ahsa-mir-877">LUAD		TCGA-44-2655-11A-01R-1757-13	TCGA-44-2657-11A-01R-1757-13	TCGA-44-2661-11A-01R-1757-13	TCGA-44-2665-11A-01R-1757-13	TCGA-44-2668-11A-01R-1757-13	TCGA-44-3396-11A-01R-1757-13	TCGA-44-3398-11B-01R-1757-13
hsa-mir-8		42.47	65.08	45.57	25.1	80.56	25.04	18.59

TCGA-44-6144-11A-01H-2169-13	TCGA-44-6776-11A-01H-2169-13	TCGA-44-6777-11A-01H-2169-13	TCGA-44-6778-11A-01H-2169-13	TCGA-44-7659-11A-01H-2065-13	TCGA-44-7660-11A-01H-2065-13	TCGA-44-7661-11A-01H-2065-13	TCGA-44-7662-11A-01H-2065-13	TCGA-44-7667-11A-01H-2065-13
1.04	1.3	1.14	0.63	0.77	1.12	0.48	0.76	0.4

Normal								
TCGA-44-7669-11A-01H-2065-13	TCGA-44-7670-11A-01H-2065-13	TCGA-44-7671-11A-01H-2065-13	TCGA-44-7672-11A-01H-2065-13	TCGA-49-6742-11A-01H-2169-13	TCGA-49-6743-11A-01H-2169-13	TCGA-49-6744-11A-01H-2169-13	TCGA-49-6745-11A-01H-2169-13	TCGA-50-5930-11A-01H-2169-13
0.83	0.63	0.68	1.14	1.6	0.89	0.48	1	0.38

TCGA-50-5932-11A-01H-2169-13	TCGA-50-5933-11A-01H-2169-13	TCGA-50-7109-11A-01H-2038-13	TCGA-55-7283-11A-01H-2038-13	TCGA-55-7570-11A-01H-2038-13	TCGA-55-7574-11A-01H-2038-13	TCGA-55-7576-11A-01H-2065-13	TCGA-55-7724-11A-01H-2169-13	TCGA-55-7725-11A-01H-2169-13
1.02	0.7	1.23	0.73	1.79	1.99	0.67	0.66	0.91

TCGA-55-7726-11A-01H-2169-13	TCGA-55-7903-11A-01H-2169-13	TCGA-55-7910-11A-01H-2169-13	TCGA-55-7911-11A-01H-2169-13	TCGA-55-7914-11A-01H-2169-13	TCGA-78-7163-11A-01H-2065-13	TCGA-78-7540-11A-01H-2065-13	TCGA-86-7711-11A-01H-2065-13	TCGA-86-7713-11A-01H-2065-13
0.41	2.58	1.26	2.04	0.29	1.21	0.5	1.6	0.8

TCGA-91-6835-11A-01H-2169-13	TCGA-91-6836-11A-01H-2169-13	TCGA-93-7348-11A-01H-2038-13		TCGA-05-4384-01A-01T-1754-13	TCGA-05-4390-01A-02T-1754-13	TCGA-05-4396-01A-21H-1857-13	TCGA-05-4405-01A-21H-1857-13	TCGA-05-4410-01A-21H-1857-13
1.68	1.32	1.06		2.14	5.08	3.76	1.53	1.31

TCGA-05-4415-01A-22H-1857-13	TCGA-05-4417-01A-22H-1857-13	TCGA-05-4424-01A-22H-1857-13	TCGA-05-4425-01A-01T-1754-13	TCGA-05-4427-01A-21H-1857-13	TCGA-05-4433-01A-22H-1857-13	TCGA-05-5420-01A-01T-1627-13	TCGA-05-5423-01A-01T-1627-13	TCGA-05-5425-01A-02T-1627-13
8.13	4.11	11.19	2.77	0.3	2.81	0.84	0.98	1.17

TCGA-05-5428-01A-01T-1627-13	TCGA-05-5429-01A-01T-1627-13	TCGA-05-5715-01A-01T-1627-13	TCGA-35-5375-01A-01T-1627-13	TCGA-38-4631-01A-01T-1754-13	TCGA-38-4632-01A-01T-1754-13	TCGA-38-6178-01A-11H-1754-13	TCGA-38-7271-01A-11H-2038-13	TCGA-38-A44F-01A-11H-A24G-13
1.06	0.56	5.12	1.84	26.6	4.06	4.76	1.4	0.32

TCGA-44-5643-01A-01T-1627-13	TCGA-44-5644-01A-21H-2038-13	TCGA-44-5645-01A-01T-1627-13	TCGA-44-6144-01A-11H-1754-13	TCGA-44-6145-01A-11H-1754-13	TCGA-44-6146-01A-11H-1754-13	TCGA-44-6146-01A-11H-A279-13	TCGA-44-6146-01B-04R-A27D-13	TCGA-44-6147-01A-11H-1754-13
3.64	11.44	1.05	10.03	2.71	8.87	4.16	6.74	4.74

TCGA-44-6147-01A-11H-A279-13	TCGA-44-6147-01B-06R-A27D-13	TCGA-44-6148-01A-11H-1754-13	TCGA-44-6774-01A-21H-1857-13	TCGA-44-6775-01A-11H-1857-13	TCGA-44-6775-01A-11H-A279-13	TCGA-44-6775-01C-02R-A27D-13	TCGA-44-6776-01A-11H-1857-13	TCGA-44-6777-01A-11H-1857-13
5.95	9.89	1.45	2.3	2	1.8	14.04	1.08	0.1

TCGA-44-6778-01A-11H-1857-13	TCGA-44-6779-01A-11H-1857-13	TCGA-44-7659-01A-11H-2065-13	TCGA-44-7660-01A-11H-2065-13	TCGA-44-7661-01A-11H-2065-13	TCGA-44-7662-01A-11H-2065-13	TCGA-44-7667-01A-31H-2065-13	TCGA-44-7669-01A-21H-2065-13	TCGA-44-7670-01A-11H-2065-13
0.93	0.1	1.02	5.84	3.5	2.36	8.11	7.91	8.47

TCGA-44-7671-01A-11H-2065-13	TCGA-44-7672-01A-11H-2065-13	TCGA-44-8117-01A-11H-2240-13	TCGA-44-8119-01A-11H-2240-13	TCGA-44-8120-01A-11H-2240-13	TCGA-44-A479-01A-31H-A24G-13	TCGA-44-A47A-01A-21H-A24G-13	TCGA-44-A47B-01A-11H-A24G-13	TCGA-44-A47F-01A-11H-A24G-13
2.25	1.85	2.6	3.32	4.97	1.56	1.29	1.41	1.9

TCGA-44-A47G-01A-21H-A24G-13	TCGA-44-A4SS-01A-11H-A24S-13	TCGA-44-A4SU-01A-11H-A24S-13	TCGA-49-4487-01A-21H-1857-13	TCGA-49-4488-01A-01T-1754-13	TCGA-49-4490-01A-21H-1857-13	TCGA-49-4512-01A-21H-1857-13	TCGA-49-4514-01A-21H-1857-13	TCGA-49-6742-01A-11H-1857-13
2.12	1.81	6.44	2.05	8.42	2.58	5.68	8.54	1.31

TCGA-49-6743-01A-11H-1857-13	TCGA-49-6744-01A-11H-1857-13	TCGA-49-6745-01A-11H-1857-13	TCGA-49-6761-01A-31H-1948-13	TCGA-49-6767-01A-11H-1857-13	TCGA-49-AAQV-01A-11H-A39B-13	TCGA-49-AAR0-01A-21H-A39B-13	TCGA-49-AAR2-01A-11H-A39B-13	TCGA-49-AAR3-01A-11H-A41D-13
2.47	1.13	0.62	2.44	3.84	2.31	3.15	10.02	2.11

TCGA-49-AAR4-01A-12H-A41D-13	TCGA-49-AAR9-01A-21H-A41D-13	TCGA-49-AARE-01A-11H-A41D-13	TCGA-49-AARN-01A-21H-A41D-13	TCGA-49-AARO-01A-12H-A41D-13	TCGA-49-AARQ-01A-11H-A41D-13	TCGA-49-AARR-01A-11H-A41D-13	TCGA-4B-A93V-01A-11H-A39B-13	TCGA-50-5044-01A-21H-1857-13
1.99	2.6	2.33	1.63	1.18	4.45	2.44	5.69	1.4

TCGA-50-5045-01A-01T-1627-13	TCGA-50-5049-01A-01T-1627-13	TCGA-50-5051-01A-21H-1857-13	TCGA-50-5055-01A-01T-1627-13	TCGA-50-5066-01A-01T-1627-13	TCGA-50-5066-02A-11H-2089-13	TCGA-50-5068-01A-01T-1627-13	TCGA-50-5072-01A-21H-1857-13	TCGA-50-5930-01A-11H-1754-13
0.66	1.39	1.87	1.88	1.41	2.65	0.74	3.13	3.17

TCGA-50-5931-01A-11H-1754-13	TCGA-50-5932-01A-11H-1754-13	TCGA-50-5933-01A-11H-1754-13	TCGA-50-5935-01A-11H-1754-13	TCGA-50-5936-01A-11H-1627-13	TCGA-50-5939-01A-11H-1627-13	TCGA-50-5941-01A-11H-1754-13	TCGA-50-5942-01A-21H-1754-13	TCGA-50-5944-01A-11H-1754-13
8.44	3.77	0.94	2.79	2.03	2.77	4.68	2.59	2.24

TCGA-50-5946-01A-11H-1754-13	TCGA-50-5946-02A-11H-2089-13	TCGA-50-6590-01A-12H-1857-13	TCGA-50-6591-01A-11H-1754-13	TCGA-50-6592-01A-11H-1754-13	TCGA-50-6593-01A-11H-1754-13	TCGA-50-6594-01A-11H-1754-13	TCGA-50-6595-01A-12H-1857-13	TCGA-50-6597-01A-11H-1857-13
5.56	11.94	2.75	42.82	1.93	2.98	1.39	2.45	2.36

TCGA-50-6673-01A-11H-1948-13	TCGA-50-7109-01A-11H-2038-13	TCGA-50-8457-01A-11H-2325-13	TCGA-50-8459-01A-11H-2325-13	TCGA-50-8460-01A-11H-2325-13	TCGA-53-7624-01A-11H-2065-13	TCGA-53-7626-01A-12H-2065-13	TCGA-53-7813-01A-11H-2169-13	TCGA-53-A4EZ-01A-12H-A24S-13
7.02	2.56	0.97	0.51	2.08	5.36	2.17	3.66	6.89

TCGA-55-5899-01A-11H-1627-13	TCGA-55-6543-01A-11H-1754-13	TCGA-55-6642-01A-11H-1857-13	TCGA-55-6712-01A-11H-1857-13	TCGA-55-6968-01A-11H-1948-13	TCGA-55-6969-01A-11H-1948-13	TCGA-55-6970-01A-11H-1948-13	TCGA-55-6971-01A-11H-1948-13	TCGA-55-6972-01A-11H-1948-13
7.29	3.36	0.81	0.85	3.68	9.5	3.23	1.54	8.07

TCGA-55-6978-01A-11H-1948-13	TCGA-55-6979-01A-11H-1948-13	TCGA-55-6980-01A-11H-1948-13	TCGA-55-6981-01A-11H-1948-13	TCGA-55-6982-01A-11H-1948-13	TCGA-55-6983-01A-11H-1948-13	TCGA-55-6984-01A-11H-1948-13	TCGA-55-6985-01A-11H-1948-13	TCGA-55-6986-01A-11H-1948-13
3	2.76	2.57	7.08	4.02	2.41	1.98	5.23	4.69

TCGA-55-6987-01A-11H-1948-13	TCGA-55-7227-01A-11H-2038-13	TCGA-55-7281-01A-11H-2038-13	TCGA-55-7283-01A-11H-2038-13	TCGA-55-7284-01B-11H-2240-13	TCGA-55-7570-01A-11H-2038-13	TCGA-55-7573-01A-11H-2038-13	TCGA-55-7574-01A-11H-2038-13	TCGA-55-7576-01A-11H-2065-13
2.52	3.06	0.88	3.54	1.84	10.88	6.38	2.27	0.87

TCGA-55-7724-01A-11H-2169-13	TCGA-55-7725-01A-11H-2169-13	TCGA-55-7726-01A-11H-2169-13	TCGA-55-7727-01A-11H-2169-13	TCGA-55-7728-01A-11H-2186-13	TCGA-55-7815-01A-11H-2169-13	TCGA-55-7816-01A-11H-2169-13	TCGA-55-7903-01A-11H-2169-13	TCGA-55-7907-01A-11H-2169-13
1.97	2.2	1.6	6.05	1.13	2.8	0.36	7.44	11.17

TCGA-55-7910-01A-11H-2169-13	TCGA-55-7911-01A-11H-2169-13	TCGA-55-7913-01B-11H-2240-13	TCGA-55-7914-01A-11H-2169-13	TCGA-55-7994-01A-11H-2186-13	TCGA-55-7995-01A-11H-2186-13	TCGA-55-8085-01A-11H-2240-13	TCGA-55-8087-01A-11H-2240-13	TCGA-55-8089-01A-11H-2240-13
9.31	2	2.29	6.38	8.81	1.67	6.85	3.61	1.32

TCGA-55-8090-01A-11H-2240-13	TCGA-55-8091-01A-11H-2240-13	TCGA-55-8092-01A-11H-2240-13	TCGA-55-8094-01A-11H-2240-13	TCGA-55-8096-01A-11H-2240-13	TCGA-55-8097-01A-11H-2240-13	TCGA-55-8203-01A-11H-2240-13	TCGA-55-8204-01A-11H-2240-13	TCGA-55-8205-01A-11H-2240-13
2.66	1.17	1.89	11.94	4.53	1.58	2.62	2.11	1.31

TCGA-55-8206-01A-11H-2240-13	TCGA-55-8207-01A-11H-2240-13	TCGA-55-8208-01A-11H-2240-13	TCGA-55-8299-01A-11H-2286-13	TCGA-55-8301-01A-11H-2286-13	TCGA-55-8302-01A-11H-2325-13	TCGA-55-8505-01A-11H-2402-13	TCGA-55-8506-01A-11H-2402-13	TCGA-55-8507-01A-11H-2402-13
2.42	6.25	3.55	2.43	3.54	1.14	4.19	1.45	9.45

TCGA-55-8508-01A-11H-2402-13	TCGA-55-8510-01A-11H-2402-13	TCGA-55-8511-01A-11H-2402-13	TCGA-55-8512-01A-11H-2402-13	TCGA-55-8513-01A-11H-2402-13	TCGA-55-8514-01A-11H-2402-13	TCGA-55-8614-01A-11H-2402-13	TCGA-55-8615-01A-11H-2402-13	TCGA-55-8616-01A-11H-2402-13
1.84	1.96	5.48	2.47	1.89	1.33	2.15	2.96	4.82

TCGA-55-8619-01A-11H-2402-13	TCGA-55-8620-01A-11H-2402-13	TCGA-55-8621-01A-11H-2402-13	TCGA-55-A48X-01A-11H-A24G-13	TCGA-55-A48Y-01A-11H-A24G-13	TCGA-55-A48Z-01A-12H-A24S-13	TCGA-55-A490-01A-11H-A24G-13	TCGA-55-A491-01A-11H-A24G-13	TCGA-55-A492-01A-11H-A24G-13
1.82	2.62	0.25	2.59	2.28	3.35	5.61	1.39	1.83

TCGA-55-A493-01A-11H-A24G-13	TCGA-55-A494-01A-11H-A24S-13	TCGA-55-A4DF-01A-11H-A24G-13	TCGA-55-A4DG-01A-11H-A24G-13	TCGA-55-A57B-01A-12H-A39B-13	TCGA-62-8394-01A-11H-2325-13	TCGA-62-8395-01A-11H-2325-13	TCGA-62-8397-01A-11H-2325-13	TCGA-62-8398-01A-11H-2325-13
4.19	7.21	3.4	6.22	4.01	4.95	1.13	2.04	3.23

TCGA-62-8399-01A-21H-2325-13	TCGA-62-8402-01A-11H-2325-13	TCGA-62-A46O-01A-11H-A24G-13	TCGA-62-A46P-01A-11H-A24G-13	TCGA-62-A46R-01A-11H-A24G-13	TCGA-62-A46S-01A-11H-A24G-13	TCGA-62-A46U-01A-11H-A24G-13	TCGA-62-A46V-01A-11H-A24G-13	TCGA-62-A46Y-01A-11H-A24G-13
3.94	6.23	2.73	1.37	0.95	4.17	2.6	2.09	7.34

								Tumor
TCGA-62-A470-01A-11H-A24G-13	TCGA-62-A471-01A-12H-A24G-13	TCGA-62-A472-01A-11H-A24G-13	TCGA-64-1679-01A-21H-2065-13	TCGA-64-1681-01A-11H-2065-13	TCGA-64-5774-01A-01T-1627-13	TCGA-64-5775-01A-01T-1627-13	TCGA-64-5778-01A-01T-1627-13	TCGA-64-5779-01A-01T-1627-13
6.52	1.33	0.94	3.04	2.5	1.24	4.44	2.38	6.76

TCGA-64-5781-01A-01T-1627-13	TCGA-64-5815-01A-01T-1627-13	TCGA-67-4679-01B-01T-1754-13	TCGA-67-6215-01A-11H-1754-13	TCGA-67-6216-01A-11H-1754-13	TCGA-67-6217-01A-11H-1754-13	TCGA-69-7760-01A-11H-2169-13	TCGA-69-7761-01A-11H-2169-13	TCGA-69-7763-01A-11H-2169-13
0.77	0.57	2.51	2.67	0.45	8.31	2.35	2.12	1.95

TCGA-69-7764-01A-11H-2169-13	TCGA-69-7765-01A-11H-2169-13	TCGA-69-7973-01A-11H-2186-13	TCGA-69-7974-01A-11H-2186-13	TCGA-69-7978-01A-11H-2186-13	TCGA-69-7979-01A-11H-2186-13	TCGA-69-7980-01A-11H-2186-13	TCGA-69-8253-01A-11H-2286-13	TCGA-69-8254-01A-11H-2286-13
1.31	3.79	2.64	1.23	2.31	12.42	5.84	1.19	3.74

TCGA-69-8255-01A-11H-2286-13	TCGA-69-8453-01A-12H-2325-13	TCGA-69-A59K-01A-11H-A263-13	TCGA-71-6725-01A-11H-1857-13	TCGA-71-8520-01A-11H-2402-13	TCGA-73-4658-01A-01T-1754-13	TCGA-73-4676-01A-01T-1754-13	TCGA-73-7498-01A-12H-2186-13	TCGA-73-7499-01A-11H-2186-13
5.84	4.3	1.89	2.66	2.16	0.57	0.93	6.57	2.17

TCGA-73-A9RS-01A-11H-A41D-13	TCGA-75-5122-01A-01T-1754-13	TCGA-75-5125-01A-01T-1754-13	TCGA-75-5126-01A-01T-1754-13	TCGA-75-5146-01A-01T-1627-13	TCGA-75-5147-01A-01T-1627-13	TCGA-75-6203-01A-11H-1754-13	TCGA-75-6205-01A-11H-1754-13	TCGA-75-6206-01A-11H-1754-13
2.35	1.29	2	1.7	0.98	3.44	1.79	3.46	4.84

TCGA-75-6207-01A-11H-1754-13	TCGA-75-6211-01A-11H-1754-13	TCGA-75-6212-01A-11H-1754-13	TCGA-75-6214-01A-41H-1948-13	TCGA-75-7025-01A-12H-1948-13	TCGA-75-7027-01A-11H-1948-13	TCGA-75-7030-01A-11H-1948-13	TCGA-75-7031-01A-11H-1948-13	TCGA-78-7143-01A-11H-2038-13
2.48	4.39	2.2	11.13	3.68	2.67	0.94	27.33	2.76

TCGA-78-7145-01A-11H-2038-13	TCGA-78-7146-01A-11H-2038-13	TCGA-78-7147-01A-11H-2038-13	TCGA-78-7148-01A-11H-2038-13	TCGA-78-7149-01A-11H-2038-13	TCGA-78-7150-01A-21H-2038-13	TCGA-78-7152-01A-11H-2038-13	TCGA-78-7153-01A-11H-2038-13	TCGA-78-7154-01A-11H-2038-13
2.23	5.88	3.88	1.71	8.07	1.75	1.01	2.45	3.08

TCGA-78-7155-01A-11H-2038-13	TCGA-78-7156-01A-11H-2038-13	TCGA-78-7158-01A-11H-2038-13	TCGA-78-7159-01A-11H-2038-13	TCGA-78-7160-01A-11H-2038-13	TCGA-78-7161-01A-11H-2038-13	TCGA-78-7162-01A-21H-2065-13	TCGA-78-7163-01A-12H-2065-13	TCGA-78-7166-01A-12H-2065-13
6.66	2.04	0.47	14.27	2.24	6.49	4.94	5.33	1.66

TCGA-78-7167-01A-11H-2065-13	TCGA-78-7220-01A-11H-2038-13	TCGA-78-7535-01A-11H-2065-13	TCGA-78-7536-01A-11H-2065-13	TCGA-78-7537-01A-11H-2065-13	TCGA-78-7539-01A-11H-2065-13	TCGA-78-7540-01A-11H-2065-13	TCGA-78-7542-01A-21H-2065-13	TCGA-78-7633-01A-11H-2065-13
3.47	3.55	3.62	4.51	2.65	1.57	1.77	3.6	3.12

TCGA-78-8640-01A-11H-2402-13	TCGA-78-8648-01A-11H-2402-13	TCGA-78-8655-01A-11H-2402-13	TCGA-78-8660-01A-11H-2402-13	TCGA-78-8662-01A-11H-2402-13	TCGA-80-5607-01A-31H-1948-13	TCGA-80-5608-01A-31H-1948-13	TCGA-80-5611-01A-01T-1627-13	TCGA-83-5908-01A-21H-2286-13
4.34	2.02	0.92	5.14	7.4	12.03	9.29	6.83	4.22

TCGA-86-6562-01A-11H-1754-13	TCGA-86-6851-01A-11H-1948-13	TCGA-86-7701-01A-11H-2169-13	TCGA-86-7711-01A-11H-2065-13	TCGA-86-7713-01A-11H-2065-13	TCGA-86-7714-01A-12H-2169-13	TCGA-86-7953-01A-11H-2186-13	TCGA-86-7954-01A-11H-2186-13	TCGA-86-7955-01A-11H-2186-13
1.56	5.81	2	6.51	4.36	1.6	2.37	5.86	6.41

TCGA-86-8054-01A-11H-2240-13	TCGA-86-8055-01A-11H-2240-13	TCGA-86-8056-01A-11H-2240-13	TCGA-86-8073-01A-11H-2240-13	TCGA-86-8074-01A-11H-2240-13	TCGA-86-8075-01A-11H-2240-13	TCGA-86-8076-01A-31H-2240-13	TCGA-86-8278-01A-11H-2286-13	TCGA-86-8279-01A-11H-2286-13
5.74	2.75	1.36	2.12	4.6	4.52	2.36	1	6.27

TCGA-86-8280-01A-11H-2286-13	TCGA-86-8281-01A-11H-2286-13	TCGA-86-8358-01A-11H-2325-13	TCGA-86-8359-01A-11H-2325-13	TCGA-86-8585-01A-11H-2402-13	TCGA-86-8668-01A-11H-2402-13	TCGA-86-8669-01A-11H-2402-13	TCGA-86-8671-01A-11H-2402-13	TCGA-86-8672-01A-21H-2402-13
2.69	1.22	12.18	1.24	3.29	1.74	0.68	2.55	1.47

TCGA-86-8673-01A-11H-2402-13	TCGA-86-8674-01A-21H-2402-13	TCGA-86-A456-01A-11H-A24G-13	TCGA-86-A4D0-01A-11H-A24G-13	TCGA-86-A4JF-01A-11H-A24S-13	TCGA-86-A4P7-01A-11H-A24S-13	TCGA-86-A4P8-01A-11H-A24S-13	TCGA-91-6828-01A-11H-1857-13	TCGA-91-6829-01A-21H-1857-13
1.34	1.98	2.19	10.88	3.7	1.88	1.8	4.31	2.69

TCGA-91-6830-01A-11H-1948-13	TCGA-91-6831-01A-11H-1857-13	TCGA-91-6835-01A-11H-1857-13	TCGA-91-6836-01A-21H-1857-13	TCGA-91-6840-01A-11H-1948-13	TCGA-91-6847-01A-11H-1948-13	TCGA-91-6848-01A-11H-1948-13	TCGA-91-6849-01A-11H-1948-13	TCGA-91-7771-01A-11H-2169-13
3.97	3.62	1.68	3.65	21.66	6.49	7.13	1.84	3.01

TCGA-91-8496-01A-11H-2402-13	TCGA-91-8497-01A-11H-2402-13	TCGA-91-8499-01A-11H-2402-13	TCGA-91-A4BC-01A-11R-A24G-13	TCGA-91-A4BD-01A-11R-A24G-13	TCGA-93-7347-01A-11H-2186-13	TCGA-93-7348-01A-21H-2038-13	TCGA-93-8067-01A-11H-2286-13	TCGA-93-A4JN-01A-11H-A24S-13
0.78	1.66	4.34	2.42	1.61	1.23	2.21	2.58	1.67

TCGA-93-A4JO-01A-21H-A24S-13	TCGA-93-A4JP-01A-11H-A24S-13	TCGA-93-A4JQ-01A-11H-A24S-13	TCGA-95-7039-01A-11H-1948-13	TCGA-95-7043-01A-11H-1948-13	TCGA-95-7562-01A-11H-2240-13	TCGA-95-7567-01A-11H-2065-13	TCGA-95-7944-01A-11H-2186-13	TCGA-95-7947-01A-11H-2186-13
0.97	5.24	4.68	3.88	2.64	13.04	3.84	0.68	1.91

TCGA-95-7948-01A-11H-2186-13	TCGA-95-8039-01A-11H-2240-13	TCGA-95-8494-01A-11H-2325-13	TCGA-95-A4VK-01A-11H-A263-13	TCGA-95-A4VN-01A-11H-A263-13	TCGA-95-A4VP-01A-21H-A263-13	TCGA-97-7546-01A-11H-2038-13	TCGA-97-7547-01A-11H-2038-13	TCGA-97-7552-01A-11H-2038-13
2.62	3.17	1.04	1.05	2.07	3.61	2.84	1.94	1.64

TCGA-97-7553-01A-21H-2038-13	TCGA-97-7554-01A-11H-2038-13	TCGA-97-7937-01A-11H-2169-13	TCGA-97-7938-01A-11H-2169-13	TCGA-97-7941-01A-11H-2186-13	TCGA-97-8171-01A-11H-2286-13	TCGA-97-8172-01A-11H-2286-13	TCGA-97-8174-01A-11H-2286-13	TCGA-97-8175-01A-11H-2286-13
1.43	3.73	2.6	1.17	1.09	4.38	1.28	2.79	1.72

TCGA-97-8176-01A-11H-2402-13	TCGA-97-8177-01A-11H-2286-13	TCGA-97-8179-01A-11H-2286-13	TCGA-97-8547-01A-11H-2402-13	TCGA-97-8552-01A-11H-2402-13	TCGA-97-A4LX-01A-11H-A24S-13	TCGA-97-A4M0-01A-11H-A24S-13	TCGA-97-A4M1-01A-11H-A24S-13	TCGA-97-A4M2-01A-12H-A24S-13
2.04	0.52	4.09	0.77	2.01	1.57	0.68	1.32	2.83

TCGA-97-A4M3-01A-11H-A24S-13	TCGA-97-A4M5-01A-11H-A24S-13	TCGA-97-A4M6-01A-11H-A24S-13	TCGA-97-A4M7-01A-11H-A24S-13	TCGA-99-7458-01A-11H-2038-13	TCGA-99-8025-01A-11H-2240-13	TCGA-99-8028-01A-11H-2240-13	TCGA-99-8032-01A-11H-2240-13	TCGA-99-8033-01A-11H-2240-13
2.9	0.64	0.98	1.88	2.55	1.71	0.96	4.07	2.39

TCGA-99-AA5R-01A-11H-A39B-13	TCGA-J2-8192-01A-11H-2240-13	TCGA-J2-8194-01A-11H-2240-13	TCGA-J2-A4AD-01A-11R-A24G-13	TCGA-J2-A4AE-01A-21R-A24G-13	TCGA-J2-A4AG-01A-11H-A24G-13	TCGA-L4-A4E5-01A-11H-A24S-13	TCGA-L4-A4E6-01A-11R-A24G-13	TCGA-L9-A443-01A-12H-A24G-13
2.32	2.43	3.88	4.67	2.64	0.91	4.48	1.93	1.25

TCGA-L9-A444-01A-21H-A24G-13	TCGA-L9-A50W-01A-12H-A39B-13	TCGA-L9-A5IP-01A-21H-A39B-13	TCGA-L9-A743-01A-43H-A39B-13	TCGA-L9-A7SV-01A-11H-A39B-13	TCGA-L9-A8F4-01A-11H-A39B-13	TCGA-MN-A4N1-01A-11H-A24S-13	TCGA-MN-A4N4-01A-12H-A24S-13	TCGA-MN-A4N5-01A-11H-A24S-13
3.29	3	0.88	2.51	1.95	3.51	3.88	1.37	2.39

TCGA-MP-A4SV-01A-11H-A24S-13	TCGA-MP-A4SW-01A-21H-A24S-13	TCGA-MP-A4SY-01A-21H-A24S-13	TCGA-MP-A4T2-01A-11H-A24S-13	TCGA-MP-A4T4-01A-11H-A263-13	TCGA-MP-A4T6-01A-32H-A263-13	TCGA-MP-A4T7-01A-11H-A24S-13	TCGA-MP-A4T8-01A-11H-A24S-13	TCGA-MP-A4T9-01A-11H-A24S-13
4.14	2.21	1.84	0.66	1.92	7.32	4.15	0.98	2.24

TCGA-MP-A4TA-01A-21H-A24S-13	TCGA-MP-A4TC-01A-11H-A24S-13	TCGA-MP-A4TD-01A-32H-A263-13	TCGA-MP-A4TE-01A-22H-A263-13	TCGA-MP-A4TF-01A-11H-A263-13	TCGA-MP-A4TH-01A-31H-A263-13	TCGA-MP-A4TI-01A-21H-A24S-13	TCGA-MP-A4TJ-01A-51H-A263-13	TCGA-MP-A4TK-01A-11H-A24S-13
8.83	2.37	1.57	3.7	4.23	5.74	1.36	2.69	2.91

TCGA-MP-A5C7-01A-11H-A263-13	TCGA-NJ-A4YF-01A-12H-A263-13	TCGA-NJ-A4YG-01A-22H-A263-13	TCGA-NJ-A4YI-01A-11H-A263-13	TCGA-NJ-A4YP-01A-11H-A263-13	TCGA-NJ-A4YQ-01A-11H-A263-13	TCGA-NJ-A55A-01A-11H-A263-13	TCGA-NJ-A55O-01A-11H-A263-13	TCGA-NJ-A55R-01A-11H-A263-13
4.37	10.95	2.22	1.27	1.76	1.15	1.68	3.57	3.12

TCGA-NJ-A7XG-01A-12H-A39B-13	TCGA-O1-A52J-01A-11H-A263-13	TCGA-S2-AA1A-01A-12H-A39B-13
0.89	9.98	1.89

Normal	Lung cancer
42.47	2.14
65.08	5.08
45.57	3.76
25.1	1.53
80.56	1.31
25.04	8.13
18.59	4.11
1.04	11.19
1.3	2.77
1.14	0.3
0.63	2.81
0.77	0.84
1.12	0.98
0.48	1.17
0.76	1.06
0.4	0.56
0.83	5.12
0.63	1.84
0.68	26.6
1.14	4.06
1.6	4.76
0.89	1.4
0.48	0.32
1	3.64
0.38	11.44
1.02	1.05
0.7	10.03
1.23	2.71
0.73	8.87
1.79	4.16
1.99	6.74
0.67	4.74
0.66	5.95
0.91	9.89
0.41	1.45
2.58	2.3
1.26	2
2.04	1.8
0.29	14.04
1.21	1.08
0.5	0.1
1.6	0.93
0.8	0.1

1.68	1.02
1.32	5.84
1.06	3.5
	2.36
	8.11
	7.91
	8.47
	2.25
	1.85
	2.6
	3.32
	4.97
	1.56
	1.29
	1.41
	1.9
	2.12
	1.81
	6.44
	2.05
	8.42
	2.58
	5.68
	8.54
	1.31
	2.47
	1.13
	0.62
	2.44
	3.84
	2.31
	3.15
	10.02
	2.11
	1.99
	2.6
	2.33
	1.63
	1.18
	4.45
	2.44
	5.69
	1.4
	0.66

1.39
1.87
1.88
1.41
2.65
0.74
3.13
3.17
8.44
3.77
0.94
2.79
2.03
2.77
4.68
2.59
2.24
5.56
11.94
2.75
42.82
1.93
2.98
1.39
2.45
2.36
7.02
2.56
0.97
0.51
2.08
5.36
2.17
3.66
6.89
7.29
3.36
0.81
0.85
3.68
9.5
3.23
1.54
8.07

3
2.76
2.57
7.08
4.02
2.41
1.98
5.23
4.69
2.52
3.06
0.88
3.54
1.84
10.88
6.38
2.27
0.87
1.97
2.2
1.6
6.05
1.13
2.8
0.36
7.44
11.17
9.31
2
2.29
6.38
8.81
1.67
6.85
3.61
1.32
2.66
1.17
1.89
11.94
4.53
1.58
2.62
2.11

1.31
2.42
6.25
3.55
2.43
3.54
1.14
4.19
1.45
9.45
1.84
1.96
5.48
2.47
1.89
1.33
2.15
2.96
4.82
1.82
2.62
0.25
2.59
2.28
3.35
5.61
1.39
1.83
4.19
7.21
3.4
6.22
4.01
4.95
1.13
2.04
3.23
3.94
6.23
2.73
1.37
0.95
4.17
2.6

2.09
7.34
6.52
1.33
0.94
3.04
2.5
1.24
4.44
2.38
6.76
0.77
0.57
2.51
2.67
0.45
8.31
2.35
2.12
1.95
1.31
3.79
2.64
1.23
2.31
12.42
5.84
1.19
3.74
5.84
4.3
1.89
2.66
2.16
0.57
0.93
6.57
2.17
2.35
1.29
2
1.7
0.98
3.44

1.79
3.46
4.84
2.48
4.39
2.2
11.13
3.68
2.67
0.94
27.33
2.76
2.23
5.88
3.88
1.71
8.07
1.75
1.01
2.45
3.08
6.66
2.04
0.47
14.27
2.24
6.49
4.94
5.33
1.66
3.47
3.55
3.62
4.51
2.65
1.57
1.77
3.6
3.12
4.34
2.02
0.92
5.14
7.4

12.03
9.29
6.83
4.22
1.56
5.81
2
6.51
4.36
1.6
2.37
5.86
6.41
5.74
2.75
1.36
2.12
4.6
4.52
2.36
1
6.27
2.69
1.22
12.18
1.24
3.29
1.74
0.68
2.55
1.47
1.34
1.98
2.19
10.88
3.7
1.88
1.8
4.31
2.69
3.97
3.62
1.68
3.65

21.66
6.49
7.13
1.84
3.01
0.78
1.66
4.34
2.42
1.61
1.23
2.21
2.58
1.67
0.97
5.24
4.68
3.88
2.64
13.04
3.84
0.68
1.91
2.62
3.17
1.04
1.05
2.07
3.61
2.84
1.94
1.64
1.43
3.73
2.6
1.17
1.09
4.38
1.28
2.79
1.72
2.04
0.52
4.09

0.77
2.01
1.57
0.68
1.32
2.83
2.9
0.64
0.98
1.88
2.55
1.71
0.96
4.07
2.39
2.32
2.43
3.88
4.67
2.64
0.91
4.48
1.93
1.25
3.29
3
0.88
2.51
1.95
3.51
3.88
1.37
2.39
4.14
2.21
1.84
0.66
1.92
7.32
4.15
0.98
2.24
8.83
2.37

1.57
3.7
4.23
5.74
1.36
2.69
2.91
4.37
10.95
2.22
1.27
1.76
1.15
1.68
3.57
3.12
0.89
9.98
1.89
