# Supplementary material for: MicroRNA-877-5p Inhibits Cell Progression by Targeting FOXM1 in Lung Cancer
Source: Can Respir J. 2022 Jun 15;2022:4256172. doi: 10.1155/2022/4256172 (PMC9217556; doi:10.1155/2022/4256172)
Supplement: Supplementary Materials — This section provides additional information about the expression of miR-877-5p in the TCGA publicly available lung cancer dataset (Supplementary Figure S1 and Supplementary Excel S1), targets, and Venn diagrams of miR-877-5p predicted by five databases cross-analysing (Supplementary Figure S2 and Supplementary Excel S2). [file 4256172.f1.zip › 4256172.f1/Supplement Excle S2.pdf]

miRDB	miRDiP	TargetScan	miRTarBase	Starbase
FGB	PLEKHA3	KIF6	RPLP1	ZFY
FOXD4L5	FOXM1	SYT7	LRRC57	CLSTN1
COL6A3	TP53INP2	COX20	SYNPR	DFFA
C15orf40	ANKFY1	PDHB	LETMD1	HSPB7
C5orf47	AR	KRAS	GYLTL1B	EPHA2
DDO	DYRK1A	LIAS	PTGFRN	EMC1
TTC14	ZNF174	CLTC	MAPRE1	LUZP1
CEP350	ELF1	IGF2BP2	NDE1	ASAP3
ATE1	KATNBL1	TMCC2	TBCC	HMGCL
DEPDC4	HAND2	ISX	TMEM167A	GPATCH3
FXR2	KRAS	CNP	CDR2L	SLC9A1
CSNK1G3	TMCC2	WDR7	EIF2AK2	WASF2
ITM2B	YLPM1	VPS41	PLK1	TMEM200B
ATP11B	FXR2	FOXP4	HMGB3	SNRNP40
VGLL3	CDC40	RFTN2	PTP4A2	C1orf216
ESRP1	SCN3A	DCX	FOXM1	C1orf109
KRTAP4-11	SORBS3	ZC3H6	NFAT5	EPHA10
KRTAP4-9	TMED7	SHISA6	RC3H1	SCMH1
ANO6	CCN5	KLHL24	SEC16A	HIVEP3
LMBRD1	HOXB8	DNMT3A	MC4R	FOXJ3
MAP7D3	ATP2B1	YPEL2	NACA	C1orf210
ETFRF1	NUP98	WWC1	ETS2	SLC6A9
KRTAP4-8	GNB4	ZBTB20	INPP4A	IPP
SORBS3	REV3L	CDC40	HSPA8	PIK3R3
C4orf33	SLCO3A1	SBNO1	RNF126	EFCAB14
ZNF174	KLHL24	KLHL18	DSCR3	EPS15
BTD	LPCAT2	TSPAN6	ARHGEF6	KTI12
BAZ1A	IGF2BP2	FKBP5	SEC24C	CC2D1B
GLDC	EID1	HNRNPC	AP3S1	SSBP3
ZFYVE26	ITM2B	FXR2	ZBTB11	SERBP1
GRK2	BAZ2B	CBFA2T3	TET1	GNG12
ZNF264	CSNK1G3	CBL	TUBB2B	WLS
CFHR5	NCOR1	ORC2	RPL35	ANKRD13C
TDRD1	KRTAP4-9	NEBL	CHAMP1	PTGER3
CDO1	MN1	KAT7	FAM72A	CTBS
ATP2B1	FKBP5	MAT1A	DDX47	SYDE2
PANK3	LAMP2	ATP2B1	TMEM106B	DBT
ELF1	BAZ1A	SNX30	INTS1	EXTL2
E2F5	RFTN2	SAP30L	RNF167	PSRC1
EPDR1	TMBIM6	FAM204A	C17orf51	SORT1
ASTN2	NEBL	CSNK1G3	ARL9	SIKE1
GALR1	NRK	SESN3	TOB2	GDAP2
LMAN1	COL6A3	SLC38A9	TUBA1B	WARS2
ANKFY1	ZBTB41	KDM5C	SHISA5	PRKAB2
STYK1	DCX	FSD1L	MEF2D	NBPF20
BTN1A1	DCAF12	OTOF	NELFE	MCL1
UBN2	ZNF280C	TENT2	STAU1	ENSA
YLPM1	UBN2	PLEKHG3	CCND2	CTSS
TRIM10	WASL	ARHGAP19	ECEL1	CERS2
ALDH5A1	KAT7	MTDH	HNRNPUL1	CDC42SE1

ZC3H12B	PCDH19	PDPN	FBXL8	RFX5
TLL2	TSPAN6	ZNF280C	RPS27	SELENBP1
PDE1C	KLHL18	SRPX2	ND1	TDRKH
DPY30	RALBP1	AAK1	RPL23A	GATAD2B
DYRK1A	PFKM	LPCAT2	CAB39	SLC39A1
GFOD2	VPS41	DHTKD1	SLC25A5	ADAR
HLA-DRA	CREBRF	RELCH	RAB1B	FAM189B
LOX	PDPN	TP53INP2	USP30	ASH1L
EEF1AKNMT	CBFA2T3	HAND2	UCHL1	SMG5
HELQ	ADAMTSL1	USP46	ANP32A	MEF2D
CTBS	ZFYVE26	METTL22	MEGF9	CRABP2
ZKSCAN2	ARHGAP19	VLDLR	TCF25	HDGF
GNB4	HNRNPC	ZNF174	TMEM30A	SLAMF6
POLQ	ZBTB20	PLPP3	MTHFD1	SLAMF1
NME4	KLHDC10	SORBS3	CHD8	APOA2
POU2F1	PHTF2	MCAM	FAM122B	SLC19A2
KTI12	CAP1	EIF1AX	USP33	RC3H1
OCSTAMP	DHTKD1	WTIP	GORASP2	STX6
CDC40	YAP1	ATXN1L	UBR4	RGS16
CDKN1B	HSD11B1	PLCE1	GLUL	EDEM3
ADAMTSL1	PHOX2B	BAZ2B	SLC25A1	ZBTB41
C14orf119	RNF111	ZNF827	USP2	PHLDA3
SKA2	FRMD3	DTWD2	PRND	KDM5B
RDX	ITCH	TCEANC2	SQSTM1	RABIF
OSTC	NR4A3	NMNAT2	PPIL4	PLEKHA6
PHF8	SMAP1	NR4A3	DGKD	NUAK2
HOXA10	EGR1	RNF152	FOXC1	IL10
CNTN3	FSD1L	UBN2	THRA	YOD1
PLEKHB1	ZNF264	SYNCRIP	FAM171A1	LPGAT1
AMER1	MACROD2	SCN3A	ERF	BATF3
TP53INP2	RPS6KA3	IL1RAPL1	CSTB	ANGEL2
FSD1L	RBM25	PLEKHA3	SIGMAR1	GPATCH2
CIP2A	ITGB8	KATNBL1	PRCC	WDR26
CAMLG	SEC63	TPST2	TMEM147	ENAH
KRAS	YPEL2	FAM167A	HIST1H2BL	ACBD3
ZNF596	ZNF827	BEND6	LRRFIP1	JMJD4
SERPINB9	DPY19L3	TMED7	LEPR	C1orf35
STARD7	LCOR	HOXB8	UBE2N	CCSAP
PCDH19	KPNA1	DCAF12	NOTCH2NL	EGLN1
STON2	ETNK1	SHISA9	SREBF2	TOMM20
INPP4A	SOBP	XKRX	MTMR3	NID1
MAPK8	MACC1	CDKN1B	GNB2L1	GREM2
RIPOR2	FOXP4	FRMD3	NR2F2	CHML
ATP7B	DICER1	YLPM1	EIF5A	RER1
GXYLT1	CELF2	SKIL	SRRM2	VAMP3
ADAMTS3	MTMR4	PBRM1	KIAA1147	H6PD
IRS2	NAV1	CD248	CAMSAP1	SPSB1
SLCO3A1	ZFYVE28	NCOR1	CASC3	NMNAT1
FBXW7	ASTN2	COL6A3	UBC	KAZN
HERC3	RC3H1	ELF1	SLC25A3	SPEN
PHIP	NSD2	FOXM1	RNF41	SZRD1

CBLL1	BTG2	CAPN7	SURF4	ELOA
KATNBL1	C18orf25	MTHFD1L	LNPEP	PNRC2
SCN3A	PRDM4	PRMT6	GRB10	CLIC4
APOBEC4	ING3	TWF2	BRD2	SELENON
WWTR1	LMBRD1	PRDM4	ZNF174	PDIK1L
CTTNBP2	PHF8	SLCO3A1	SAP18	CEP85
KLHL24	WDFY1	KRTAP4-9	KMT2D	DHDDS
DPY19L3	CBL	AOX1	MPI	PPP1R8
GPATCH2	BRINP3	HSD11B1	MARCKS	XKR8
CCN5	RAC1	BAMBI	PLD3	ATP5IF1
MACC1	ENC1	PFKM	ATP5O	YTHDF2
HAND2	ZC3H6	ANKFY1	HIST1H3B	KPNA6
AGL	ESRP1	PROSER2	AKAP1	RBBP4
WDR31	CDKN1B	DPY19L3	MLXIP	S100PBP
DDX52	REEP1	ADAMTSL1	HUWE1	ZMYM1
MN1	RUBCN	ELOA	TXN2	AGO4
SLC22A13	SYNCRIP	SIGLEC5	WBP11	AGO1
LACTB	KMT2C	POLH	PHPT1	ZC3H12A
FOXD4	ORAI1	NRK	MAPK8	CDCA8
KPNA1	WWC1	CCN5	PTDSS1	AKIRIN1
GABRG1	TCEANC2		ELF4	CAP1
CCDC34	ISX		TUBB	EXO5
KLHL18	PHF3		TAF11	CTPS1
TSPAN31	CAMLG		RPS6KB1	NSUN4
PDCL3	STAMBP		SLC39A1	CMPK1
ARFRP1	CEP350		ZNF280B	CDKN2C
ZNRF1	HLA-DRA		ZKSCAN8	TCEANC2
NDUFA8	NMNAT2		ACBD3	PRKAA2
RABGAP1L	MAN1A1		AGO1	NFIA
TMEM144	ORC2		OTUD7B	HS2ST1
ALCAM	ZYG11B		UBL5	LRRC8C
IKZF4	IRS2		AFF1	AGL
RNF212B	ADAR		ZBTB38	MFSD14A
ETNK1	INPP4A		PSMD11	AC118553.2
FOXD4L1	STAG2		IGF2	PRMT6
FRMD3	RUFY3		ADCY3	STXBP3
TMEM196	PHIP		TMX3	CSF1
FOXD4L3	NIPA1		NDUFB11	DCLRE1B
SNX30	AUTS2		ZNF283	OLFML3
FOXD4L6	NRP2		CMTM8	PTGFRN
STRAP	EIF1AX		NUCKS1	MAN1A2
TRPA1	RP11-204N11.1		RNASEK	FAM72B
BMPR2	GABRG2		ND2	SEC22B
ZPLD1	AMOT		PAPOLA	RNF115
DENND6A	POU2F1		FAM127A	NBPF12
GRIP2	PCMTD1		DNAJB9	PIP5K1A
ZNF626	PIKFYVE		PHLPP1	ZNF687
ITCH	SLF2		TEX264	S100A9
RAC1	PDE4D		CENPF	SNAPIN
MMP16	MAP7D3		SORBS3	UBAP2L
GABRA4	AAK1		PROX1	RUSC1

STXBP3	MAP2K4		SMCHD1	LMNA
PTH2R	ALG9		SMG1	DUSP23
PAIP1	AMD1		PI4K2A	PEA15
ZBTB41	NEO1		ACTA1	UFC1
PALM2	CLTC		ACTG1	UHMK1
VPS13C	EMP1		IGF2BP2	RGS4
PHTF2	HERC3		MTMR14	POGK
AFF3	RCHY1		LARP1	TBX19
STAMBP	SPRED1		TPP1	PRRX1
KLHDC10	ASXL1		THRAP3	PRRC2C
CDCA8	SNX30		ORC2	METTL13
ABHD10	ELAVL1		HADH	TOR1AIP1
RUBCN	IL1RAP		TMEM184B	QSOX1
STK39	ANTXR1		ATP5G3	XPR1
ATP8A1	SBNO1		HMGA1	MR1
TMCC2	AOX1		VDAC1	IER5
SHB	ANKRD13C		FBXO7	LAMC1
SP8	PEG10		EEF1A1	C1orf21
GPX1	KTI12		HAND1	SWT1
KLF8	ATXN1L		MICU1	ODR4
GOLGA1	SHB		CCNT2	NR5A2
LRRC32	RUNX1T1		SH3GL1	CAMSAP2
CABLES1	KAT6B		SRSF3	NAV1
GNRHR	PRRC2C		BTG2	IPO9
LMBRD2	DTD2		GINS4	PPP1R12B
LPCAT2	UTRN		PNRC2	BTG2
ESYT3	LNPK		BTF3L4	ATP2B4
BCL2L13	C15orf40		PGRMC1	ZC3H11A
GLIPR1	PANK3		YAP1	ZBED6
RNASE7	SLC25A36		VAMP8	SOX13
KCNJ15	RFX5		PCNA	FAM72A
STAC	ZNRF1		GNL1	SRGAP2
BCCIP	KLF8		THEM6	RASSF5
ZNF615	SHISA6		ZFP91	PFKFB2
TMEM50B			YOD1	CD46
ZNF69			WASL	HSD11B1
NUFIP1			RPS24	DIEXF
DCX			NCBP2	HHAT
UPRT			DNAJB4	ATF3
BLMH			CDKN1B	CENPF
TMBIM6			CD4	C1orf115
DHTKD1			AMOTL2	MARC1
GSPT1			AFF2	MIA3
EMP1			KRAS	FBXO28
FOXM1			ZSCAN25	ARF1
SMAP1			C12orf4	IBA57
PTPRG			MRPL51	RHOU
ZBTB20			MED18	URB2
PIK3AP1			BCL2L2	ZNF672
IKZF1			HOXA10	ASB13
KCNA1			EIF1AX	GDI2

FAM161A			SERF2	NMT2
REEP1			STOML1	MKX
PAK2			CRCP	KIF5B
CUX1			SPHAR	ZNF33B
CNTLN			RAB4A	CXCL12
TMEM178B			HYPK	8-Mar
ELAC2			CASP16	ZFAND4
AR			SLC35E2	ZWINT
RNF168			ZNF566	IPMK
THEM6			COLEC10	SLC16A9
SLC25A21			CELF1	CCDC6
PLEKHA3			CECR2	RUFY2
GNAZ			AGO3	DNA2
NDUFA4			COLEC12	AIFM2
KCTD18			ARHGDIA	LRRC20
HOXB8			HSPA12B	ANXA7
FGF14			TMEM170B	USP54
DLG2			DNAH10OS	NDST2
CLEC5A			RNF8	AC022400.6
GON7			RELA	AP3M1
CD302			FAM213A	PPP1R3C
SYNPO			GATSL2	FGFBP3
NAV1			TTC28	IDE
ZNF326			CYB561	PIK3AP1
LY75-CD302			AQP3	ARHGAP19
SLF2			CYB561	MORN4
TENT5A			TMEM170B	CHUK
ZNF519			TTC28	NDUFB8
SUCNR1			RELA	POLL
ZYG11B			GATSL2	MGEA5
NDST3			TMEM170B	LDB1
GCSAM			FAM213A	SH3PXD2A
MMRN1			RELA	GPAM
GRAMD2A			RNF8	CCDC186
ZNF280C			DNAH10OS	RAB11FIP2
KRIT1			TMEM170B	PRDX3
TRMT6			HSPA12B	CHST15
ZNF440			CDKN1B	OAT
AMD1			MRPL51	FAM53B
IMPACT			ARHGDIA	CELF2
PHF14			HOXA10	DHTKD1
IGF1R			CDKN1B	PTER
HGF			VAMP8	ARL5B
MAN1A1			YAP1	MAP3K8
YAP1			COLEC12	CCDC7
VKORC1L1			EIF1AX	ZNF33A
PTK7			HOXA10	MAPK8
C10orf111			PGRMC1	ARID5B
ASB11			PNRC2	ZNF365
IL2RA			PCNA	EIF4EBP2
PNPO			GINS4	ANAPC16

ZNF14			COLEC12	VCL
CACNA2D2			EIF1AX	KAT6B
XBP1			PCNA	ZMIZ1
XDH			AGO3	FAM213A
DCAF12			CDKN1B	FAS
HSP90AA1			CECR2	IFIT2
ADCY2			CELF1	TNKS2
TP53I11			COLEC10	5-Mar
CD44			ZNF566	EXOC6
ZPR1			SLC35E2	SLC35G1
RFTN2			BCL2L2	SLF2
FZD5			CASP16	TWNK
ODAPH			EIF1AX	NOLC1
BROX			HOXA10	MFSD13A
VPS41			HYPK	TRIM8
ITGB6			RAB4A	WBP1L
KSR2			SPHAR	ADD3
PXK			MED18	ADRA2A
SC5D			CRCP	TDRD1
UNC13C			STOML1	FAM160B1
BCL2L15			SERF2	TRUB1
SERF2			CDKN1B	INPP5F
GTF3C4			ZSCAN25	PLEKHA1
NCOR1			EIF1AX	BCCIP
OCRL			HOXA10	SIRT3
CCDC174			BCL2L2	GATD1
MEGF9			MED18	OSBPL5
PAX9			PCNA	NUP98
SKIL			MRPL51	TRIM3
PTCHD4			C12orf4	ARFIP2
OGA			ZSCAN25	TPP1
GALNT10			GINS4	DENND5A
MAPK8IP2			KRAS	EIF4G2
DKK2			THEM6	PLEKHA7
BTG2			AFF2	E2F8
SPRED1			PCNA	SVIP
HTR2A			AFF2	CCDC34
NRG4			PCNA	CSTF3
TREM1			AMOTL2	SLC1A2
TM4SF1			BTF3L4	TRAF6
PRPS2			BTG2	TP53I11
ZNF346			CD4	CELF1
NSF			CDKN1B	PATL1
UTS2			DNAJB4	TMEM223
ITGB8			NCBP2	MEN1
ZNF91			RNF126	FOSL1
ZDHHC15			RPS24	EIF1AD
CLEC7A			WASL	SPTBN2
VWA5A			YOD1	ALDH3B2
EPB41L5			ZFP91	KMT5B
HAUS6			THEM6	FAM168A

TOR1AIP1			SRSF3	XRRA1
EHMT1			GNL1	CCDC90B
SEC22A			PCNA	RAB38
FKBP5				CTSC
SERINC3				SESN3
GCFC2				MMP1
MYLK4				MSANTD4
TMEM132B				KDELC2
FAM83A				PPP2R1B
SMG5				ALG9
BAZ2B				CADM1
DDX6				ZPR1
GDPD1				DPAGT1
IRF1				MCAM
GDAP1L1				NECTIN1
C22orf46				ZNF202
NIPA1				SRPRA
A1CF				MUC5B
ZNF365				STIM1
APLNR				TIMM10B
PKHD1				TUB
SCUBE3				RPL27A
RBM25				PARVA
MAP3K19				TEAD1
ZNF33A				C11orf58
AIFM2				NCR3LG1
AFTPH				CAPRIN1
ADARB2				EHF
ARHGAP19				CD44
ANTXR1				TRIM44
ZNF597				LDLRAD3
ZNF585B				API5
NMNAT1				SLC35C1
AQP3				ATG13
NFATC2				SLC39A13
IPMK				CLP1
PEG10				ZDHHC5
NRK				SELENOH
RHOQ				AP001931.1
ASXL3				CTNND1
CACNA1C				ZFP91
ARRDC4				TKFC
ACHE				ESRRA
GABRG2				PPP2R5B
CXorf67				SNX15
CTPS1				ARL2-SNX15
ZNF689				DPF2
SEPHS2				BANF1
ZNF317				PACS1
				RAB1B
				RBM14

				KDM2A
				GRK2
				PPP6R3
				CTTN
				RNF121
				ARHGEF17
				PLEKHB1
				RNF169
				TSKU
				TMEM126B
				TMEM135
				PIWIL4
				FUT4
				AMOTL1
				SRSF8
				C11orf70
				YAP1
				AASDHPPT
				DIXDC1
				DLAT
				RBM7
				SIDT2
				TAGLN
				ARCN1
				HINFP
				NLRX1
				CBL
				VWA5A
				FLI1
				APLP2
				VPS26B
				KDM5A
				FOXM1
				C12orf4
				SCNN1A
				MRPL51
				ZNF384
				C1RL
				RERG
				KRAS
				GXYLT1
				SCAF11
				SLC38A1
				SLC38A2
				HDAC7
				ARF3
				RHEBL1
				LMBR1L
				LIMA1
				FIGNL2
				KRT80

				SPRYD3
				RARG
				ANKRD52
				TIMELESS
				B4GALNT1
				PPM1H
				KRR1
				LIN7A
				ATP2B1
				TMCC3
				NTN4
				ARL1
				GNPTAB
				SLC41A2
				PRDM4
				GIT2
				NAA25
				RASAL1
				WSB2
				GCN1
				KDM2B
				RSRC2
				SBNO1
				SCARB1
				SLC15A4
				DDX51
				POLE
				ERC1
				ADIPOR2
				LTBR
				CD4
				ATN1
				NECAP1
				GABARAPL1
				CDKN1B
				APOLD1
				DDX47
				GPRC5A
				FAM234B
				EMP1
				STRAP
				PDE3A
				ETNK1
				ETFRF1
				STK38L
				KLHL42
				FAR2
				ALG10B
				PPHLN1
				IRAK4
				TMEM117

				PFKM
				SPATS2
				KCNH3
				TMBIM6
				SMARCD1
				DIP2B
				LETMD1
				ACVRL1
				NR4A1
				ATG101
				EIF4B
				HOXC11
				HOXC8
				COPZ1
				RAB5B
				IKZF4
				MYL6
				SPRYD4
				TSPAN31
				SLC16A7
				SRGAP1
				TBK1
				DYRK2
				FRS2
				THAP2
				NAV3
				MRPL42
				METAP2
				TDG
				UBE3B
				PTPN11
				OAS3
				OAS2
				RITA1
				RNF10
				MLEC
				ORAI1
				BCL7A
				EP400
				XPO4
				RNF6
				FOXO1
				ELF1
				KBTBD7
				NUFIP1
				INTS6
				ATP7B
				VPS36
				DIAPH3
				KCTD12
				ABCC4

				STK24
				IRS2
				COL4A1
				PAN3
				FREM2
				SLC25A15
				AKAP11
				ITM2B
				TRIM13
				CKAP2
				BIVM
				ERCC5
				BIVM-ERCC5
				ING1
				CUL4A
				TMCO3
				HNRNPC
				CHD8
				RAB2B
				RBM23
				PSMB5
				ZFHX2
				SDR39U1
				DTD2
				EGLN3
				BAZ1A
				RALGAPA1
				CLEC14A
				MAP4K5
				NID2
				GNPNAT1
				DDHD1
				EXOC5
				GPR135
				SIX4
				RAB15
				MAX
				ZFYVE26
				ELMSAN1
				AREL1
				PGF
				TGFB3
				VIPAS39
				ALKBH1
				GTF2A1
				GALC
				GON7
				BTBD7
				DICER1
				ATG2B
				BCL11B

				SLC25A29
				HSP90AA1
				ARHGEF40
				ABHD4
				LRP10
				BCL2L2
				NGDN
				THTPA
				DCAF11
				PSME1
				NPAS3
				FAM177A1
				KIAA0391
				AL121594.1
				PAX9
				LRFN5
				KLHDC2
				GNG2
				MAPK1IP1L
				DAAM1
				PPM1A
				PRKCH
				AKAP5
				FNTB
				SLC39A9
				SUSD6
				TTC9
				SIPA1L1
				RBM25
				FCF1
				YLPM1
				DLST
				CALM1
				UBR7
				OTUB2
				PPP2R5C
				RCOR1
				TNFAIP2
				EIF5
				TRMT61A
				KLC1
				AL139300.1
				ZFYVE21
				INF2
				NIPA1
				KATNBL1
				SLC12A6
				ZNF770
				DPH6
				FSIP1
				RMDN3

				RHOV
				ZNF106
				LCMT2
				CATSPER2
				SHC4
				SECISBP2L
				GABPB1
				ARPP19
				ALDH1A2
				RORA
				CA12
				IGDCC3
				INTS14
				CLN6
				ITGA11
				MYO9A
				ULK3
				SCAMP2
				ETFA
				PEAK1
				HOMER2
				C15orf40
				WDR73
				MFGE8
				PEX11A
				SELENOS
				TUBGCP5
				SPRED1
				DISP2
				RTF1
				SERF2
				EID1
				DTWD1
				RNF111
				C2CD4A
				LACTB
				RAB8B
				SNX1
				ZNF609
				THSD4
				BBS4
				NEO1
				ARID3B
				CLK3
				DNAJA4
				IREB2
				HYKK
				FAM103A1
				ZNF592
				AKAP13
				ABHD2

				ZNF710
				IQGAP1
				BLM
				FURIN
				NR2F2
				ARRDC4
				IGF1R
				RPUSD1
				CASKIN1
				PRSS22
				ZNF200
				ADCY9
				CORO7-PAM16
				PAM16
				USP7
				GSPT1
				RRN3
				MARF1
				SMG1
				EARS2
				ZKSCAN2
				SEPHS2
				ZNF689
				ZNF629
				NUDT21
				CMTM4
				KCTD19
				ATP6V0D1
				SLC7A6OS
				CHTF8
				VAC14
				GLG1
				RFWD3
				GINS2
				CBFA2T3
				CHMP1A
				FANCA
				NME4
				PIGQ
				RAB40C
				CACNA1H
				JPT2
				TRAF7
				PDPK1
				ZNF174
				GLIS2
				MGRN1
				PMM2
				ATF7IP2
				RMI2
				SHISA9

				ITPRIPL2
				VPS35L
				ANKS4B
				EEF2K
				TAOK2
				FBRS
				SRCAP
				GPT2
				CHD9
				FTO
				ARL2BP
				KATNB1
				PDP2
				CBFB
				SLC9A5
				THAP11
				EDC4
				PLA2G15
				HAS3
				SNTB2
				NFAT5
				WWP2
				ATXN1L
				ZNRF1
				MON1B
				KIAA0513
				FOXF1
				MAP1LC3B
				CDT1
				ZNF276
				ABR
				MYO1C
				ANKFY1
				UBE2G1
				SLC25A11
				DERL2
				ZBTB4
				FXR2
				VAMP2
				MYH10
				ELAC2
				NCOR1
				FAM222B
				FLOT2
				PHF12
				BLMH
				RFFL
				RAD51D
				ACACA
				DDX52
				PIP4K2B

				MED1
				PGAP3
				MED24
				STAT5B
				VAT1
				ATXN7L3
				DCAKD
				PLEKHM1
				KANSL1
				ZNF652
				SPAG9
				MBTD1
				SUPT4H1
				MTMR4
				SKA2
				CYB561
				LIMD2
				SMARCD2
				TEX2
				SLC16A6
				GGA3
				H3F3B
				RNF157
				CBX4
				NPLOC4
				ARHGDIA
				RAB40B
				DPH1
				OVCA2
				SGSM2
				PAFAH1B1
				RAP1GAP2
				PLD2
				USP6
				RPAIN
				RNASEK
				MPDU1
				WRAP53
				EFNB3
				KDM6B
				CYB5D1
				MAP2K4
				COX10
				AC098850.3
				RAI1
				SMCR8
				EPN2
				ALDH3A2
				MAP2K3
				WSB1
				NLK

				TMEM97
				TMEM199
				TRAF4
				ERAL1
				TAOK1
				RNF135
				NF1
				SUZ12
				ZNF207
				CDK5R1
				LASP1
				RAPGEFL1
				FKBP10
				CNP
				CNTNAP1
				RUNDC1
				RND2
				TMUB2
				NSF
				GOSR2
				PNPO
				IGF2BP1
				KAT7
				RSAD1
				TOM1L1
				STXBP4
				MSI2
				PPM1E
				YPEL2
				CLTC
				METTL2A
				TANC2
				DCAF7
				PSMC5
				PRKCA
				BPTF
				KCTD2
				SEC14L1
				BIRC5
				ENGASE
				CBX2
				GAA
				RNF213
				ENDOV
				HGS
				FOXK2
				YES1
				LPIN2
				PTPN2
				ESCO1
				TMEM241

				DSC3
				ZNF24
				EPG5
				ATP5F1A
				SMAD2
				ZBTB7C
				TCF4
				LMAN1
				CCBE1
				CDH19
				MBP
				ANKRD12
				TWSG1
				RALBP1
				RAB31
				RNMT
				CABLES1
				IMPACT
				MAPRE2
				PIK3C3
				C18orf25
				ONECUT2
				SERPINB5
				SERPINB8
				MBD3
				MKNK2
				TIMM13
				DIRAS1
				AES
				SH3GL1
				DPP9
				SLC25A23
				ELAVL1
				ZNF562
				CDC37
				TMED1
				RAB3D
				ZNF823
				PRKACA
				ASF1B
				BRD4
				AKAP8
				FKBP8
				GDF1
				CERS1
				ZNF14
				ZNF626
				ZNF91
				HSPB6
				ZNF566
				ZNF585A

				ZNF573
				C19orf47
				C19orf54
				ZNF235
				IRF2BP1
				NOVA2
				STRN4
				ZC3H4
				ZNF615
				ZNF28
				ZNF154
				ZNF417
				HCN2
				PTBP1
				CCDC94
				FEM1A
				KDM4B
				ZNF317
				MRPL4
				ZNF627
				ZNF791
				RNASEH2A
				THSD8
				ZSWIM4
				ZNF333
				SYDE1
				FAM32A
				OCEL1
				PGPEP1
				KLHL26
				ARMC6
				ZNF85
				ZNF726
				ZNF254
				DPY19L3
				FAAP24
				HAUS5
				ZNF420
				SPINT2
				MRPS12
				PLD3
				RAB4B-EGLN2
				EGLN2
				AXL
				CCDC97
				ZNF574
				ZNF225
				VASP
				CALM3
				CYTH2
				PTOV1

				ATF5
				RPL28
				ZNF264
				ZIK1
				ZNF211
				ZNF776
				ZNF587
				ZNF584
				ZNF324B
				PXDN
				MBOAT2
				WDR35
				SDC1
				APOB
				OTOF
				CCDC121
				DHX57
				SOS1
				SLC8A1
				COX7A2L
				PREPL
				BCL11A
				FAM161A
				SPRED2
				TGFA
				TEX261
				MOB1A
				TGOLN2
				ZNF514
				STARD7
				TMEM127
				ACTR1B
				LONRF2
				CHST10
				SLC35F5
				CCDC93
				BIN1
				MAP3K2
				WDR33
				FAM168B
				NCKAP5
				PRPF40A
				BAZ2B
				CD302
				STK39
				WIPF1
				LNPK
				TTC30A
				STK17B
				SATB2
				KCTD18

				ORC2
				TRAK2
				RAPH1
				INO80D
				FZD5
				IGFBP5
				WDFY1
				DOCK10
				SLC16A14
				COL6A3
				PER2
				THAP4
				SOX11
				ASAP2
				GEN1
				RAB10
				TMEM214
				SNX17
				GPN1
				BABAM2
				PPP1CB
				WDR43
				MSH6
				SPTBN1
				EHBP1
				AFTPH
				CEP68
				ANTXR1
				MXD1
				STAMBP
				MAT2A
				VAMP8
				MRPL35
				KCNIP3
				CNNM3
				INPP4A
				GCC2
				ZC3H6
				INHBB
				CCNT2
				KIF5C
				GALNT13
				TANC1
				DCAF17
				CYBRD1
				CDCA7
				PLEKHA3
				SSFA2
				FAM171B
				C2orf88
				NABP1

				FZD7
				BMPR2
				NRP2
				FASTKD2
				CREB1
				RPE
				RHBDD1
				AGAP1
				SCLY
				UBE2F-SCLY
				ASB1
				AL121758.1
				SRXN1
				IDH3B
				LZTS3
				PCNA
				TRMT6
				TMX4
				APMAP
				PLAGL2
				E2F1
				PXMP4
				NDRG3
				ZHX3
				SERINC3
				TP53RK
				B4GALT5
				SPATA2
				TMEM189-UBE2V1
				UBE2V1
				NFATC2
				ZNF217
				BCAS1
				CYP24A1
				PRELID3B
				YTHDF1
				ARFRP1
				SAMD10
				STK35
				ATRN
				PYGB
				POFUT1
				ASXL1
				ITCH
				MAP1LC3A
				TP53INP2
				PHF20
				EPB41L1
				HNF4A
				TTPAL
				WISP2

				KCNK15
				YWHAB
				STK4
				SLC2A10
				NCOA3
				ARFGEF2
				SLC9A8
				RBM38
				RAB22A
				LSM14B
				GID8
				SLC2A4RG
				NRIP1
				N6AMT1
				SYNJ1
				TMEM50B
				RUNX1
				VPS26C
				BRWD1
				PRDM15
				C2CD2
				WDR4
				SLC19A1
				LSS
				IFNAR2
				AP000295.1
				IFNAR1
				IFNGR2
				SLC5A3
				DYRK1A
				RRP1B
				PDXK
				PFKL
				ADARB1
				DGCR2
				CLTCL1
				ZNF280B
				ZNF70
				CHCHD10
				HPS4
				MN1
				XBP1
				ASCC2
				LIF
				OSM
				PIK3IP1
				PRR14L
				RBFOX2
				TXN2
				RAC2
				CARD10

				JOSD1
				CBX7
				MKL1
				TOB2
				NFAM1
				BCL2L13
				ZNF74
				SNAP29
				CRKL
				HIC2
				PPIL2
				SLC2A11
				CCDC117
				ZNRF3
				FBXO7
				MCM5
				PDXP
				Z83844.3
				Z83844.1
				TRIOBP
				MICALL1
				APOBEC3C
				MIEF1
				ATF4
				TNRC6B
				XPNPEP3
				PPP6R2
				ADM2
				MAPK8IP2
				RAD18
				MRPS25
				TBC1D5
				SLC4A7
				AZI2
				TMPPE
				CLASP2
				EPM2AIP1
				ZNF445
				CDCP1
				FYCO1
				SETD2
				SHISA5
				UQCRC1
				CELSR3
				GMPPB
				HYAL2
				TUSC2
				CISH
				AC097637.1
				TWF2
				BAP1

				PBRM1
				FAM208A
				DENND6A
				TMF1
				FOXP1
				RYBP
				CGGBP1
				ABI3BP
				CBLB
				CIP2A
				USF3
				NAA50
				GSK3B
				LRRC58
				FSTL1
				POLQ
				GOLGB1
				KPNA1
				HEG1
				SNX4
				SLC41A3
				MGLL
				H1FX
				TMCC1
				AMOTL2
				ANAPC13
				DZIP1L
				GK5
				XRN1
				PLOD2
				TM4SF1
				SSR3
				CCNL1
				IFT80
				PHC3
				EIF5A2
				PLD1
				TNFSF10
				NCEH1
				GNB4
				TMEM41A
				IGF2BP2
				ETV5
				MASP1
				TFRC
				NCBP2
				BDH1
				EDEM1
				SETD5
				MTMR14
				JAGN1

				BRK1
				VHL
				TATDN2
				TSEN2
				MKRN2
				CCDC174
				NR2C2
				BTD
				UBE2E1
				TGFBR2
				GPD1L
				FBXL2
				MYD88
				XYLB
				ACVR2B
				WDR48
				SLC25A38
				ZNF619
				ZNF197
				KLHL18
				ZNF589
				ARIH2
				PDE12
				SLMAP
				RPP14
				PTPRG
				KBTBD8
				MITF
				C3orf38
				COL8A1
				ALCAM
				ABHD10
				C3orf52
				ADPRH
				NR1I2
				ATP2C1
				FOXL2NB
				U2SURP
				SEC62
				SKIL
				ATP11B
				DVL3
				SENP2
				IL1RAP
				CCDC50
				OPA1
				FBXO45
				PAK2
				SENP5
				ZFYVE28
				MFSD10

				AFAP1
				SEL1L3
				BEND4
				ATP8A1
				USP46
				PPAT
				UBA6
				RCHY1
				G3BP2
				CNOT6L
				ANTXR2
				RASGEF1B
				WDFY3
				PYURF
				FAM13A
				GPRIN3
				TRMT10A
				CCNA2
				ANKRD50
				MFSD8
				INPP4B
				OTUD4
				SLC10A7
				FBXW7
				1-Mar
				AADAT
				SORBS2
				NSD2
				RNF4
				HTT
				PCDH7
				ATP10D
				EREG
				THAP6
				ODAPH
				SEPT11
				PRDM8
				AFF1
				HERC6
				HERC3
				SGMS2
				OSTC
				SEC24B
				ANK2
				METTL14
				FGF2
				ABHD18
				LARP1B
				C4orf33
				GAB1
				ARFIP1

				FGB
				CEP44
				TENM3
				SNX25
				SLC9A3
				LMBRD2
				C5orf42
				LIFR
				SLC38A9
				PDE4D
				MRPS27
				ENC1
				GFM2
				LHFPL2
				MTX3
				TMEM167A
				GLRX
				EPB41L4A
				REEP5
				TMED7
				DTWD2
				LOX
				RAPGEF6
				AC008695.1
				IRF1
				KIF3A
				AFF4
				HNRNPA0
				NDUFA2
				SPRY4
				TNIP1
				ADAM19
				PANK3
				FBXW11
				UBTD2
				STC2
				MXD3
				SRD5A1
				FAM105A
				OTULIN
				NPR3
				C5orf51
				GHR
				ISL1
				ITGA2
				MTREX
				MAP1B
				TNPO1
				FCHO2
				F2R
				AGGF1

				SCAMP1
				ARSK
				RGMB
				WDR36
				DCP2
				AP3S1
				CSNK1G3
				PDLIM4
				SLC22A5
				UBE2B
				SEC24A
				CAMLG
				DDX46
				C5orf24
				FAM53C
				EGR1
				UBE2D2
				CYSTM1
				EIF4EBP3
				ANKHD1-EIF4EBP3
				HARS2
				ZMAT2
				PCDHA6
				PCDHA9
				PCDHA8
				PCDHA7
				PCDHA5
				PCDHA4
				PCDHA2
				PCDHA1
				PCDHA13
				PCDHAC2
				PCDHAC1
				PCDHA11
				PCDHA10
				PCDHA12
				PCDHA3
				GRPEL2
				SLC26A2
				TCOF1
				NDST1
				GALNT10
				LARP1
				ITK
				CYFIP2
				HMMR
				WWC1
				CREBRF
				ARL10
				FAF2
				N4BP3

				SERPINB9
				TUBB2A
				F13A1
				SSR1
				TXNDC5
				BLOC1S5-TXNDC5
				ATXN1
				KIF13A
				ZKSCAN4
				CDSN
				ATF6B
				LEMD2
				FKBP5
				SRPK1
				CCND3
				ZNF318
				AARS2
				MCM3
				ICK
				GCLC
				LMBRD1
				SLC17A5
				PHIP
				FAM46A
				PGM3
				SYNCRIP
				LYRM2
				BACH2
				BVES
				PREP
				ATG5
				SEC63
				OSTM1
				ZBTB24
				REV3L
				TSPYL1
				GOPC
				MAN1A1
				SERINC1
				ARHGAP18
				LRP11
				SERAC1
				DYNLT1
				EZR
				AGPAT4
				RPS6KA2
				BPHL
				GCNT2
				E2F3
				ALDH5A1
				BTN3A2

				BTN3A1
				ZSCAN26
				ATAT1
				C6orf136
				TUBB
				DDR1
				HLA-DRA
				UHRF1BP1
				ANKS1A
				KCTD20
				SRSF3
				CDKN1A
				FOXP4
				AL365205.1
				TOMM6
				PPP2R5D
				KLC4
				PTK7
				TJAP1
				POLH
				RUNX2
				EFHC1
				TMEM14A
				FBXO9
				PHF3
				SMAP1
				SH3BGRL2
				PNRC1
				PM20D2
				UFL1
				LIN28B
				PRDM1
				SOBP
				FOXO3
				CEP57L1
				CDC40
				AMD1
				RNF217
				VTA1
				ADGRG6
				UTRN
				TAB2
				CCDC170
				ARID1B
				TULP4
				AFDN
				FAM120B
				TBP
				EIF2AK1
				CYTH3
				NDUFA4

				AGR2
				MACC1
				CYCS
				HOXA10
				HOXA11
				JAZF1
				NOD1
				DPY19L1
				KIAA0895
				ELMO1
				VPS41
				NUDCD3
				PURB
				TNS3
				GRB10
				SBDS
				BAZ1B
				BCL7B
				POM121C
				HIP1
				CACNA2D1
				CDK6
				SAMD9L
				PON1
				EPHB4
				NAPEPLD
				WASL
				PODXL
				CHCHD3
				CNOT4
				MTPN
				CREB3L2
				ZC3HAV1
				SLC37A3
				ZNF746
				KMT2C
				LMBR1
				ESYT2
				CHST12
				RAC1
				TMEM106B
				ITGB8
				SP4
				CREB5
				UBE2D4
				PPIA
				CCM2
				IKZF1
				VKORC1L1
				KCTD7
				POM121

				ELN
				CLIP2
				GTF2IRD1
				RHBDD2
				MDH2
				PHTF2
				CROT
				STEAP2
				CDK14
				ANKIB1
				PEG10
				ZNF655
				ZKSCAN1
				COPS6
				AGFG2
				CUX1
				LRRC17
				HBP1
				BCAP29
				DLD
				FOXP2
				MDFIC
				ING3
				SND1
				AHCYL2
				KLHDC10
				EXOC4
				CALD1
				TMEM140
				TRIM24
				UBN2
				LUC7L2
				CASP2
				ARHGEF5
				ZNF398
				NOM1
				DNAJB6
				SOX7
				AC105001.2
				FAM167A
				LONRF1
				DLC1
				MTUS1
				ASAH1
				PSD3
				NUDT18
				EGR3
				STC1
				NEFL
				PNMA2
				SARAF

				RNF122
				BRF2
				NSD3
				KAT6A
				THAP1
				PCMTD1
				TCEA1
				PLAG1
				IMPAD1
				SLCO5A1
				TPD52
				ZNF704
				ZFAND1
				MMP16
				RBM12B
				TP53INP1
				YWHAZ
				TAF2
				DSCC1
				HAS2
				ZHX1
				ASAP1
				PTK2
				JRK
				PLEC
				SHARPIN
				ZNF596
				CLN8
				XPO7
				SORBS3
				C8orf58
				CCAR2
				RHOBTB2
				PPP2R2A
				RBPMS
				ERLIN2
				DDHD2
				TACC1
				GOLGA7
				GINS4
				BHLHE22
				SGK3
				PI15
				ZC2HC1A
				E2F5
				TMEM67
				ESRP1
				SDC2
				VPS13B
				EBAG9
				MAL2

				FAM91A1
				ZNF572
				CHRAC1
				THEM6
				HSF1
				ERMP1
				GLDC
				PTPRD
				NFIB
				BAG1
				AQP3
				NOL6
				DCAF12
				MYORG
				FAM219A
				RPP25L
				RNF38
				PTAR1
				TMEM2
				TLE1
				SPTLC1
				FBP1
				PTCH1
				ZNF367
				ZNF782
				TSTD2
				TBC1D2
				ABCA1
				SUSD1
				FKBP15
				MEGF9
				FBXW2
				ZBTB6
				DENND1A
				GOLGA1
				PPP6C
				FAM129B
				PTRH1
				TOR2A
				AK1
				ST6GALNAC6
				AL157935.2
				ZER1
				SPOUT1
				UCK1
				ENTPD2
				KANK1
				UNC13B
				GLIPR2
				POLR1E
				GCNT1

				PSAT1
				CTSL
				PHF2
				NCBP1
				NR4A3
				STX17
				RNF20
				RAD23B
				HSDL2
				SNX30
				SLC31A1
				RGS3
				ZNF618
				COL27A1
				PAPPA
				MRRF
				ZBTB34
				RALGPS1
				SLC2A8
				DNM1
				ABL1
				PRRC2B
				TMEM141
				EHMT1
				PRKX
				ANOS1
				MID1
				GPM6B
				PIGA
				CTPS2
				EIF1AX
				RPS6KA3
				TAB3
				SLC35A2
				FAM156A
				KDM5C
				SMC1A
				PHF8
				AMER1
				EDA2R
				SNX12
				ZMYM3
				PCDH19
				TSPAN6
				TSC22D3
				ACSL4
				AMOT
				LAMP2
				ZNF280C
				RTL8B
				MAP7D3

				ARHGEF6
				MECP2
				WWC3
				HCCS
				PRPS2
				MOSPD2
				CA5B
				REPS2
				TSPAN7
				USP9X
				ZNF81
				FAM156B
				TSPYL2
				TSR2
				ZXDB
				MSN
				AR
				IGBP1
				SLC16A2
				UPRT
				PGK1
				APOOL
				FAM133A
				RPL36A
				ARMCX1
				NXT2
				SLC6A14
				DOCK11
				PGRMC1
				UBE2A
				ZBTB33
				XIAP
				OCRL
				SLC9A6
				HMGB3
				VMA21
				ZNF275
				ABCD1
				GDI1
				FUNDC2
