# Supplementary figures and images for: MicroRNA-877-5p Inhibits Cell Progression by Targeting FOXM1 in Lung Cancer
Source: Can Respir J. 2022 Jun 15;2022:4256172. doi: 10.1155/2022/4256172 (PMC9217556; doi:10.1155/2022/4256172)

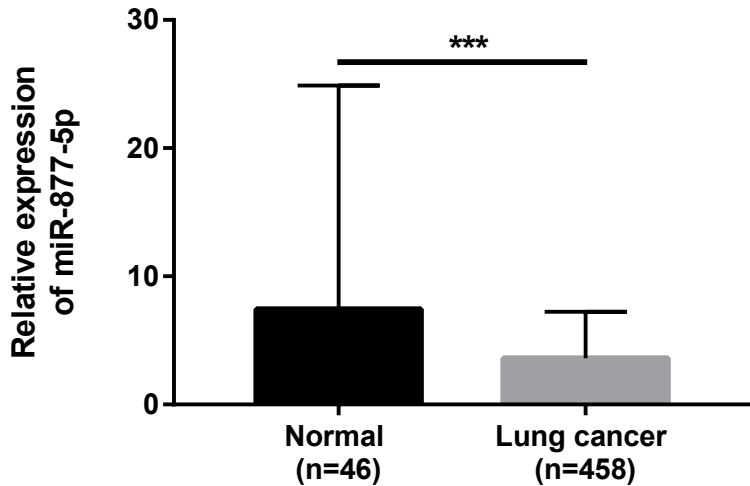

Supplement: Supplementary Materials — This section provides additional information about the expression of miR-877-5p in the TCGA publicly available lung cancer dataset (Supplementary Figure S1 and Supplementary Excel S1), targets, and Venn diagrams of miR-877-5p predicted by five databases cross-analysing (Supplementary Figure S2 and Supplementary Excel S2). [file 4256172.f1.zip › 4256172.f1/Supplementary Figure S1.pdf]

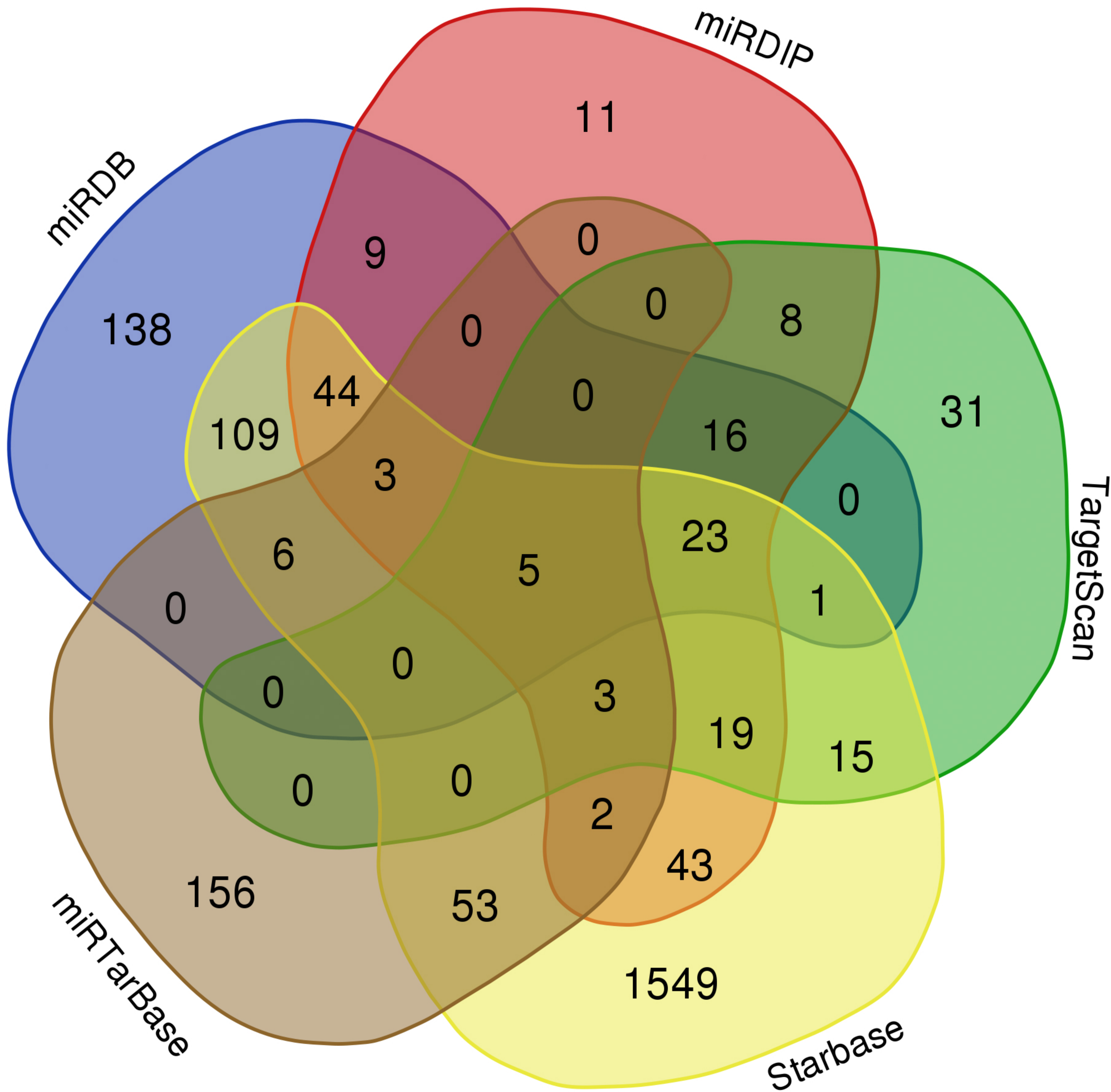

Supplement: Supplementary Materials — This section provides additional information about the expression of miR-877-5p in the TCGA publicly available lung cancer dataset (Supplementary Figure S1 and Supplementary Excel S1), targets, and Venn diagrams of miR-877-5p predicted by five databases cross-analysing (Supplementary Figure S2 and Supplementary Excel S2). [file 4256172.f1.zip › 4256172.f1/Supplementary Figure S2.pdf]
